# Convergent structural features of respiratory syncytial virus neutralizing antibodies and plasticity of the site V epitope on prefusion F

**DOI:** 10.1371/journal.ppat.1008943

**Published:** 2020-11-02

**Authors:** Wayne Harshbarger, Sai Tian, Newton Wahome, Ankita Balsaraf, Deep Bhattacharya, Desheng Jiang, Ratnesh Pandey, Kunal Tungare, Kristian Friedrich, Nurjahan Mehzabeen, Marco Biancucci, Diana Chinchilla-Olszar, Corey P. Mallett, Ying Huang, Zihao Wang, Matthew James Bottomley, Enrico Malito, Sumana Chandramouli

**Affiliations:** GSK, Rockville, MD, United States of America; Washington University in Saint Louis School of Medicine, UNITED STATES

## Abstract

Respiratory syncytial virus (RSV) is a global public health burden for which no licensed vaccine exists. To aid vaccine development via increased understanding of the protective antibody response to RSV prefusion glycoprotein F (PreF), we performed structural and functional studies using the human neutralizing antibody (nAb) RSB1. The crystal structure of PreF complexed with RSB1 reveals a conformational, pre-fusion specific site V epitope with a unique cross-protomer binding mechanism. We identify shared structural features between nAbs RSB1 and CR9501, elucidating for the first time how diverse germlines obtained from different subjects can develop convergent molecular mechanisms for recognition of the same PreF site of vulnerability. Importantly, RSB1-like nAbs were induced upon immunization with PreF in naturally-primed cattle. Together, this work reveals new details underlying the immunogenicity of site V and further supports PreF-based vaccine development efforts.

## Author summary

Respiratory syncytial virus (RSV) is a persistent, contagious seasonal pathogen and a serious public health threat. While infants are the most at-risk population, with infections potentially leading to bronchiolitis, adults, especially the elderly, are also burdened by RSV-induced respiratory infections. The only treatment currently available for RSV is passive immunization for high-risk infants. Thus, there is a critical need to develop a vaccine for the vast majority of the vulnerable population for which there is no preventative treatment. The RSV fusion protein in its prefusion form (PreF) is the target of the majority of naturally-induced neutralizing antibodies, and several clinical trials are currently evaluating PreF as a promising vaccine candidate. In this study, we solved the X-ray structure of PreF bound to the Fab fragment of a human neutralizing antibody. The structure reveals plasticity of the epitope, as well as a unique molecular signature for antibodies elicited towards this region of PreF. We also find that similar antibodies are induced upon immunization of naturally-primed cattle with a PreF vaccine antigen, suggesting that this epitope is highly immunogenic. These results will help us better understand the human immune response to RSV infection and vaccination, and guide future vaccine-design efforts.

## Introduction

Human respiratory syncytial virus (RSV) is a highly contagious member of the Pneumoviridae family of negative-sense, enveloped, RNA viruses. Over 80% of the population is exposed by the age of 2 years, making RSV among the most common causes of acute lower respiratory tract illness leading to hospitalization in children under 5 years of age [1, 2]. The disease severity and risk of hospitalization is further amplified in very young infants below the age of 6 months [3]. RSV also poses a significant health burden to older adults, often compounded by co-morbidities and an aging immune system. Currently, the only approved intervention against RSV, capable of reducing RSV-associated hospitalizations in young infants, is prophylactic administration of the monoclonal antibody (mAb) palivizumab [4]. However, due to its moderate effectiveness, high cost and the need for monthly intramuscular injections [5], its use is restricted to high-risk infants. Thus, there remains an important unmet medical need for an effective vaccine against RSV to protect all vulnerable populations [6–8]. Although several RSV vaccine programs have begun clinical development in the last decade [9], to date there is no approved vaccine [10, 11].

The RSV genome encodes 11 proteins, two of which, surface proteins F and G, are the major targets of neutralizing and non-neutralizing antibodies. RSV can be further divided into subtypes A and B which co-circulate at approximately the same rate [12]. Subtype differences are based on antigenic and genetic variability of the G protein, whereas F maintains greater than 90% sequence identity between groups [12, 13]. RSV F, a class I viral fusion protein responsible for fusing the viral and host-cell membranes [14], is the target of palivizumab and the majority of neutralizing antibodies (nAb) raised by natural infection [15–17]. RSV F is synthesized as a single chain inactive precursor (F_0_) which becomes activated upon cleavage by a furin-like protease into subunits F1 and F2, releasing a 27-amino acid glycopeptide called pep27[18]. The mature F protein undergoes large-scale conformational changes during viral entry and, upon insertion of its fusion peptide (N-terminus of F1) into the host-cell membrane, it transitions from a compact metastable prefusion state (PreF) to an elongated, energetically favorable postfusion conformation (PostF)[14]. Several past vaccine development efforts focused on PostF as the vaccine antigen due to its high stability and the presence of at least two well-characterized neutralizing sites on its surface, including the epitope of palivizumab. Unfortunately, limited efficacy of these PostF-based vaccines in protecting against RSV-associated disease has been observed in the clinic [11, 19–21].

A momentous breakthrough in the RSV vaccine field has been the structure-based design of a stabilized PreF antigen achieved through protein engineering by adding disulfide bonds and cavity-filling mutations [22, 23]. The antigen termed DS-Cav1, the most immunogenic from the original study, has been shown to elicit high nAb titers in naïve mice and primates, and in naturally-primed cattle [17, 22]. More recently, immunization of healthy human subjects with DS-Cav1 has been shown to elicit superior nAb responses as compared to historical RSV subunit vaccines [24], and several vaccine development programs that use DS-Cav1, or other variants of PreF as the main antigen, are currently ongoing [11].

PreF is the primary target of nAbs induced during natural infection and is capable of depleting nearly all the protective anti-RSV antibodies in human sera [25]. Several studies of human sera and RSV-specific human nAbs have revealed epitopes on F, allowing the identification of at least four distinct “antigenic sites”. Some of these sites are shared between PreF and PostF conformations, such as site II (bound by palivizumab and Motavizumab) and site IV (bound by mAb 101F)[15, 26–28]. Sites that are exclusively present on PreF, such as Ø and V, are known to be targeted by more potently neutralizing antibodies [29–31]. The mAbs D25, AM22, and RSD5 have been structurally characterized and found to bind to antigenic site Ø at the apex of PreF [22, 32], while mAbs CR9501 and hRSV90 have been shown to target antigenic site V which is adjacent to site Ø [30, 33]. Collectively, sites Ø and V are the targets of the majority of the human B cell repertoire for RSV [25, 34].

A comprehensive structural understanding of the immunogenic elements of these neutralizing sites will guide development of vaccine antigens that maintain the necessary features to elicit a broad and protective response. Therefore, we undertook structural and biochemical studies of the PreF variant DS-Cav1 bound to a PreF-specific antibody, RSB1. The human mAb RSB1 was identified from the serum of a naturally infected donor and showed strong competition with mAb D25[35]. Here we solved the X-ray crystal structure of the RSB1 Fab in complex with DS-Cav1, showing that RSB1 recognizes antigenic site V, and also revealing structural similarities and key differences in binding modes with the recently reported antibody CR9501. Specifically, this study identifies a nAb-induced fit to PreF, as well as a molecular signature in the RSB1 and CR9501 HCDR3 and LCDR1 regions. This work broadens our understanding of the antigenicity of site V, and the molecular basis for RSV neutralization by these nAbs.

## Results

### Neutralization, epitope binning, and HDX-MS epitope mapping of mAb RSB1

**Fig 1:**
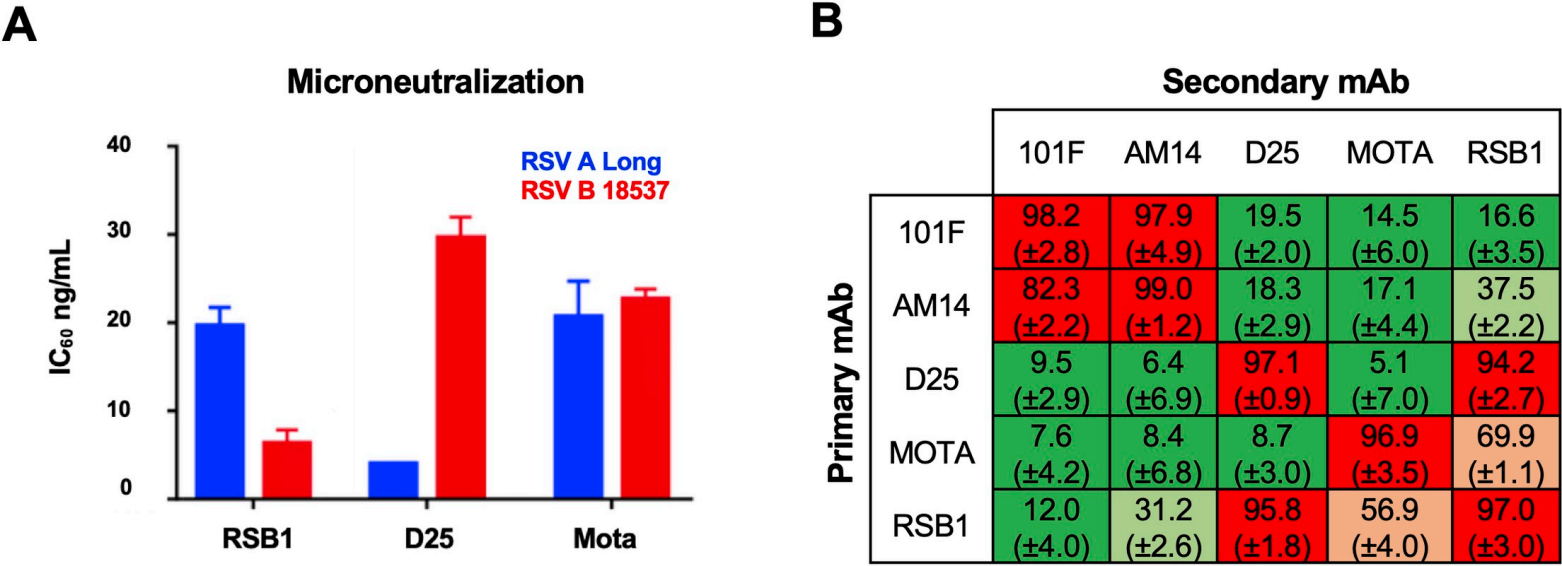
RSB1 neutralization and competition with other monoclonal antibodies. **A)** Neutralization of RSV A and B strains in a plaque reduction assay by RSB1, D25 and Motavizumab (Mota). IC_60_ neutralization titer is shown with bars colored blue for RSV strain A, and red for strain B. Error bars represent standard error based on triplicate experiments. **B)** Biolayer interferometry competition assay. The table is colored based on the percentage of competition of the primary mAbs over the secondary mAbs: green 0–29%, light green 30–49%, light orange 50–69% and red 70–100%. Numbers in parenthesis represent standard deviation from three independent experiments.

RSB1 was previously selected from a naturally infected donor in a memory B cell screening assay that identified a panel of PreF-specific neutralizing antibodies against RSV A, and based on binding competition, was grouped along with site Ø binding antibodies such as D25[35]. To further investigate the neutralization breadth of RSB1, we used a microneutralization assay to compare strains RSV A Long and RSV B18537, representing subgroups of RSV A and B, respectively. We found that RSB1 neutralizes both subtypes with high potency, though, distinct from previously reported PreF-specific nAbs, has a preference for RSV B (IC_60_ of 6 ng/mL against RSV B compared to 18 ng/mL against RSV A) (**Fig 1A)**. In contrast, D25 showed a preference for RSV A, in agreement with previous studies [36], and Motavizumab, which binds pre- and postfusion F, neutralized each virus equivalently (**Fig 1A**).

To further characterize the epitope targeted by RSB1, we performed an antibody competition assay using biolayer interferometry. The data confirmed that RSB1 competes efficiently with D25, showing greater than 90% binding competition (**Fig 1B**). However, we also discovered 70% competition with Motavizumab (site II), and 30–40% competition with AM14 which targets a PreF-specific inter-protomer epitope. This unusual competition pattern led us to utilize hydrogen deuterium exchange mass spectrometry (HDX-MS) to further map the RSB1 epitope. We found reduced deuterium exchange upon RSB1 mAb binding on two peptides in the F1 subunit [residues 161–171 (EGEVNKIKSAL) and 199–204 (IDKQLL)], and two peptides of the F2 subunit [residues 57–61 (ITIEL) and 93–96 (LQLL)] (**S1 Fig**). These peptides are only partially overlapping with the D25 epitope at site Ø (located at the apex of PreF)[23], suggesting that competition between RSB1 and D25 is more likely due to steric clashes or partially overlapping epitopes rather than binding to the same site.

### X-ray crystal structure of the DS-Cav1-RSB1 complex

**Fig 2:**
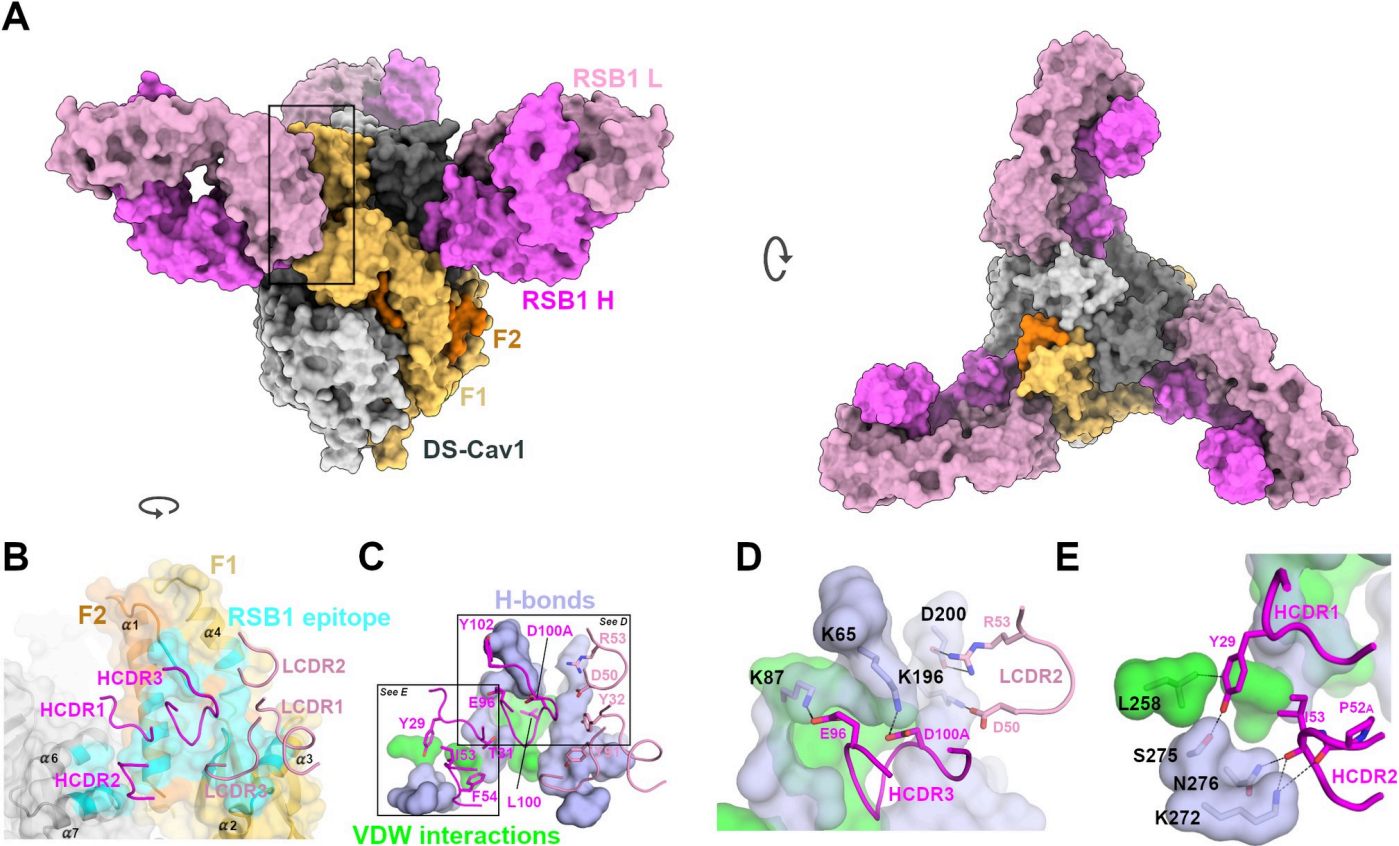
X-ray structure of the DS-Cav1-RSB1 complex. **A)** The structure of the DS-Cav1-RSB1 complex is depicted with surfaces colored in light and dark gray for two DS-Cav1 protomers, and orange and gold for the F2 and F1 subunits of the third protomer. Fab RSB1 is depicted with pink and magenta surfaces for the L and H chains, respectively. **B)** Zoomed view of the region boxed in A), after a rotation of ~90 degrees around the *y* axis. RSB1 CDR loops are depicted as cartoons and labelled. The total RSB1 epitope surface is colored cyan. **C)** Same view as in B) with the epitope hydrogen bonding residues on DS-Cav1 colored purple and the residues making van der Waals contacts colored green. RSB1 CDR residues making contacts with the purple and green regions are shown as sticks. **D)** Zoomed view of the RSB1 epitope on DS-Cav1, to highlight salt bridge interactions (sticks and dashes) only. For clarity, this view is slightly re-oriented with respect to panels B and C. **E)** Rotated and zoomed view of panel C highlighting RSB1 cross-protomer interactions with DS-Cav1 (dashes).

To fully elucidate the RSB1 paratope and the targeted PreF epitope architecture and better understand the competition with multiple antigenic sites, we determined the crystal structure of the DS-Cav1-RSB1 Fab complex, as well as the RSB1 Fab Apo structure, at 3.7 Å and 2.0 Å resolution, respectively (**Fig 2**) (**S1 Table**). Well-ordered electron densities in the complex structure, especially in the region of the DS-Cav1-RSB1 interface, allowed confident building, refinement, and interpretation of the antibody/antigen interactions (**S2 Fig)**. The specificity of RSB1 for the PreF conformation rather than PostF is structurally explained by the epitope contacts becoming dramatically distally rearranged in PostF, with PreF elements transitioning between 50–100 Å in the post-fusion conformation (**S3 Fig**).

**Fig 3:**
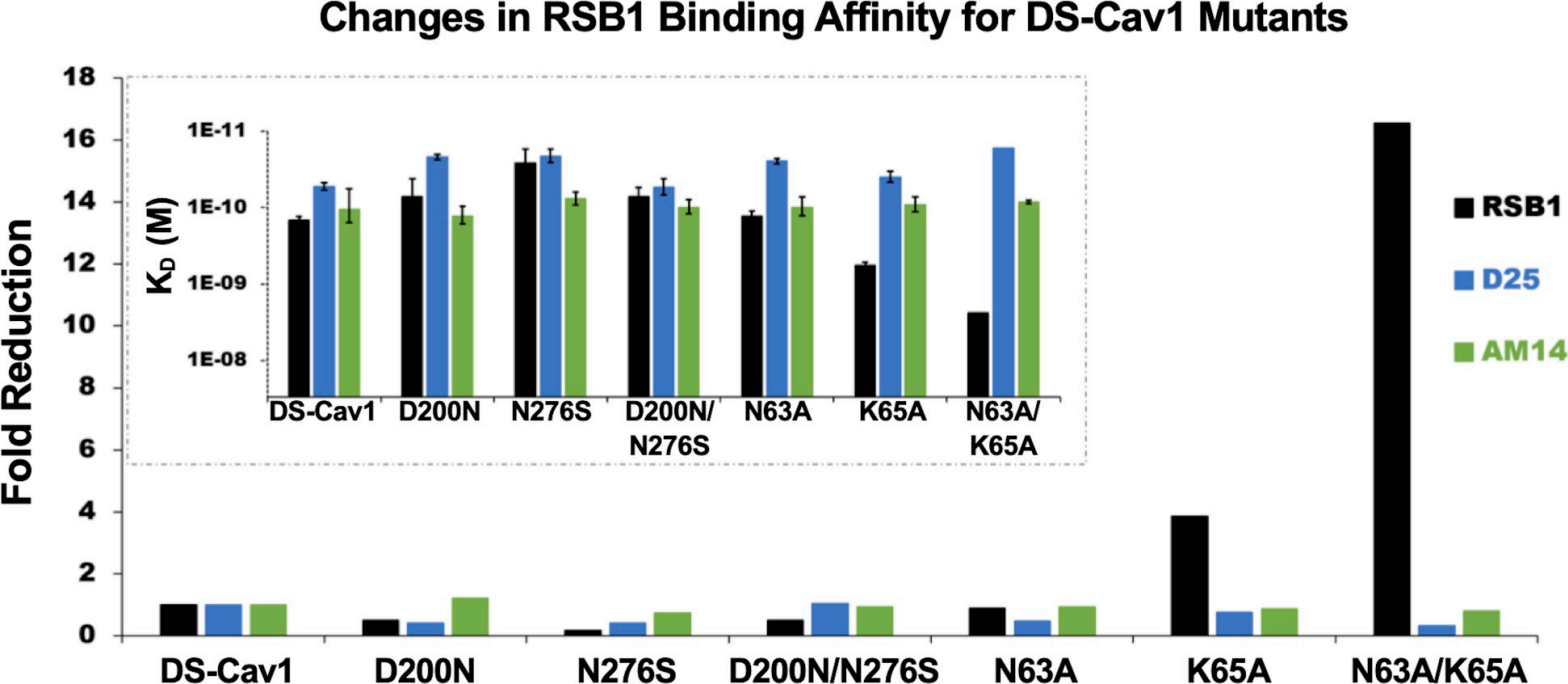
Experimental RSB1 binding affinities to DS-Cav1 and its mutants. Surface Plasmon Resonance (SPR) experiments to analyze binding affinity of RSB1, D25, and AM14 antibodies upon mutation of identified critical residues shown as fold change, with corresponding K_D_ graph shown as an inset. There is a decrease in RSB1 binding due to the single point mutation K65A and the double point mutation N63A/K65A. No change in affinity is observed with the introduction of the RSV B substitutions D200N or N276S.

In agreement with our hypothesis based on the biochemical data, we found that RSB1 mostly recognizes antigenic site V [33, 37], making light chain contacts with DS-Cav1 helices α2–3 on the F1 subunit, while also recognizing portions of F2 and site Ø on helix α1 with the heavy chain. RSB1 engages the use of all six complementarity determining regions (CDRs) to contact an extensive surface on DS-Cav1 (buried surface area ~1060 Å^2^), consistent with its high binding affinity (**Fig 3**). The Fab light chain CDR (LCDR) loops exclusively contact the F1 subunit of a single protomer and occupy a surface area of ~320 Å^2^. However, the heavy chain CDRs (HCDRs) contact both F1 and F2 of one protomer (~575 Å^2^), as well as F1 of a second protomer (~170 Å^2^), thus generating a cross-protomer paratope/epitope interaction (see below).

The HCDR3 projects its hydrophobic tip into a central, partially buried core on DS-Cav1, forming van der Waals (vdWs) contacts through Leu100 (**Fig 2C**). Importantly, the overall RSB1 epitope is highly conserved across RSV strains (**S4 Fig**) and is delimited by protruding and peripheral residues that anchor the Fab through strong salt-bridges. Specifically, Lys65 and Lys87 on F1 form salt bridges with RSB1 HCDR3 residues Glu96 and Asp100A (Kabat numbering), while F2 residues Lys196 and Asp200 form salt bridges with LCDR2 residues Asp50 and Arg53, respectively (**Fig 2D)**.

A notable feature of the RSB1 epitope is the cross-protomeric contacts to residues Leu258, Ser275, Asn276, and Lys272 (**Fig 2B and 2E**). The most prominent RSB1 cross-protomer interaction uses HCDR1 residue Tyr29 which is situated in a pocket between the two protomers, composed on one side by Leu95 (α1) of the main protomer, and on the opposite side by Leu258 (α6) of the adjacent protomer. Tyr29 also forms a hydrogen bond with Ser275 (α7) of the second protomer, while the HCDR2 backbone completes the interactions through hydrogen bonds with residues Lys272 and Asn276, both on α7 (**Fig 2E**). These interactions explain the observed competition with Motavizumab, as the helix-turn-helix feature between residues Ser255 and Val278 that comprises the Motavizumab binding site resides within the vicinity of these cross protomer contacts (**S5 Fig**). However, these contacts to four cross-protomer residues are not extensive enough to make RSB1 specific for trimeric DS-Cav1, as RSB1 binds with similarly high affinity to a monomeric form of PreF **(S2 Table)**. Therefore, it can be deduced that sufficient binding energy is achieved through the described HCDR3 and extensive salt-bridging contacts to the main DS-Cav1 protomer, whereas a more evenly distributed buried surface of the antibody between two protomers, such as that seen for AM14, is necessary for trimeric PreF-specific binding [38].

### Molecular basis for breadth of RSB1 neutralization

A single amino acid substitution between RSV A and B subtypes has been shown to be sufficient in causing subtype selectivity in the neutralization potency of the mouse monoclonal antibody 5C4 [39]. RSB1 shows high potency in neutralizing both RSV A and B strains, despite two amino acid substitutions within the otherwise highly conserved epitope (**S4 Fig**). One of these substitutions is at Asp200, which, as described above, forms a salt bridge with RSB1, and the other substitution is with the cross protomer contact Ser276. To rationalize the molecular bases of the RSB1 neutralization breadth, and to confirm binding to either subtype, we generated DS-Cav1 single and double mutants encompassing each or both of these RSV B substitutions and measured binding to RSB1 by SPR. Consistent with the neutralization data, as well as RSB1 not being trimer specific, RSB1 bound the Asn276Ser mutant without any appreciable loss in binding affinity (**Fig 3**). This is likely due to the conservation of a small polar, uncharged side chain for both A and B strains, thus maintaining a similar environment for recognition by RSB1. On the other hand, the Asp200Asn substitution would likely result in the loss of a salt bridge with RSB1 Arg53_LCDR2_ (**Fig 2D**), and comparison with the RSV B structure (PDB 6Q0S)[40] indicates there would be a shift in the local surface potential from a negatively charged region into a more neutral or positive surface (**S6 Fig**). This would seemingly disfavor binding to the RSB1 positively charged Arg53_LCDR2_. However, we found that the single mutant Asp200Asn, as well as the double mutant Asp200Asn-Asn276Ser, were again not sufficient to impact the binding affinity for RSB1. Collectively, these results support the ability for RSB1 to recognize this highly conserved epitope on DS-Cav1 and provides evidence that cross-neutralization can occur despite minor variations in the epitope.

### RSB1 structural rearrangements between bound and unbound states

Comparison of the RSB1 structures, unbound and in complex with DS-Cav1, revealed a root mean square deviation (rmsd) of 0.75 Å over 235 aligned C_α_ atoms, thus indicating little global conformational rearrangements are necessary to bind DS-Cav1 (**S7A Fig)**. Despite the similarity in overall structural conformation, two side chain re-orientations do occur between the two states. The first is the RSB1 HCDR1 residue Tyr29 (**S7B Fig)**, which as described earlier is a key component of the cross protomer interactions and is notably shifted by ~ 4 Å relative to the bound state. The second change, LCDR2 residue Arg53, is also shifted by ~ 4 Å and repositions to form the salt bridge with Asp200 in the bound structure (**S7C Fig)**. Together, these minor local induced-fit events support a relevance for these interactions with DS-Cav1.

### RSB1 and D25 bind distinct epitopes

**Fig 4:**
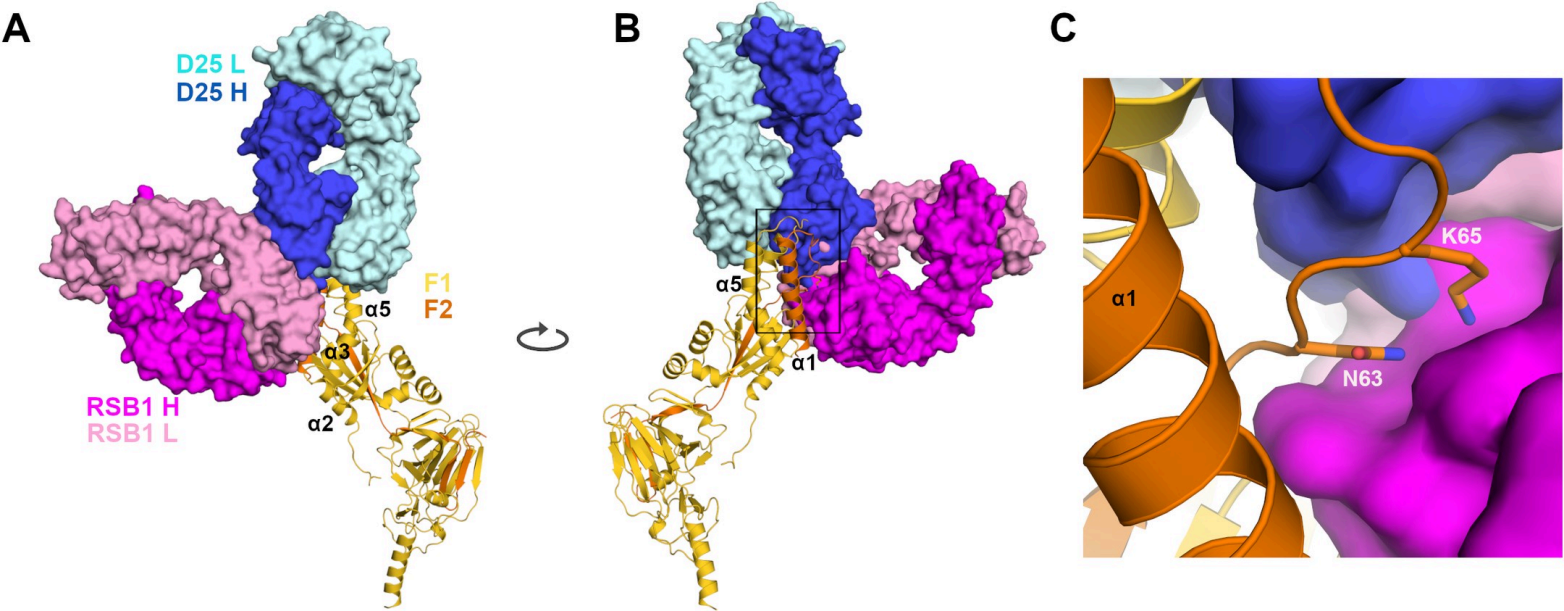
Structural comparison between DS-Cav1-RSB1 and DS-Cav1-D25 complexes. **A)** RSB1 is depicted as dark and light pink surfaces, D25 in blue and cyan. **B)** Rotated view of A. **C)** Zoomed view of boxed region in B reveals the location of residues Asn63 and Lys65, depicted as sticks.

It has been reported that site V and site Ø antibodies show competition due to the close proximity of their epitopes on PreF [30, 37]. We investigated the molecular basis for competition between D25 and RSB1 by analyzing the paratope/epitope interactions for each complex. Our analyses revealed how the RSB1 and D25 epitopes overlap only at residues Asn63 and Lys65, on helix α1 (**Fig 4**). To determine if these residues may represent an antigenic hot spot required for binding of either antibody, and to validate the RSB1 epitope identified in this study, we generated the DS-Cav1 single mutants Asn63Ala, Lys65Ala, and the double mutant Asn63Ala-Lys65Ala. While the Asn63Ala mutant did not confer a significant change in binding affinity to RSB1, as measured by SPR, there was a nearly 4-fold reduction in the binding affinity of the Lys65Ala mutant, and greater than 16-fold drop in affinity for the double mutant compared to DS-Cav1 (**Fig 3**). By contrast, no significant impact on the binding affinity of these mutants to D25 or AM14 (measured as a control) was observed. As the mutations to Asn63 and Lys65 do not affect D25 binding, RSB1 and D25 seemingly bind neighboring epitopes with distinct molecular determinants of interaction. The competition for binding likely arises from steric clashes between the antibody variable fragments (**Fig 4A–4C**) rather than engagement of a specific antigenic hot spot spanning the side chains of Asn63 and Lys65.

### Induced fit for antibodies binding Site V

**Fig 5:**
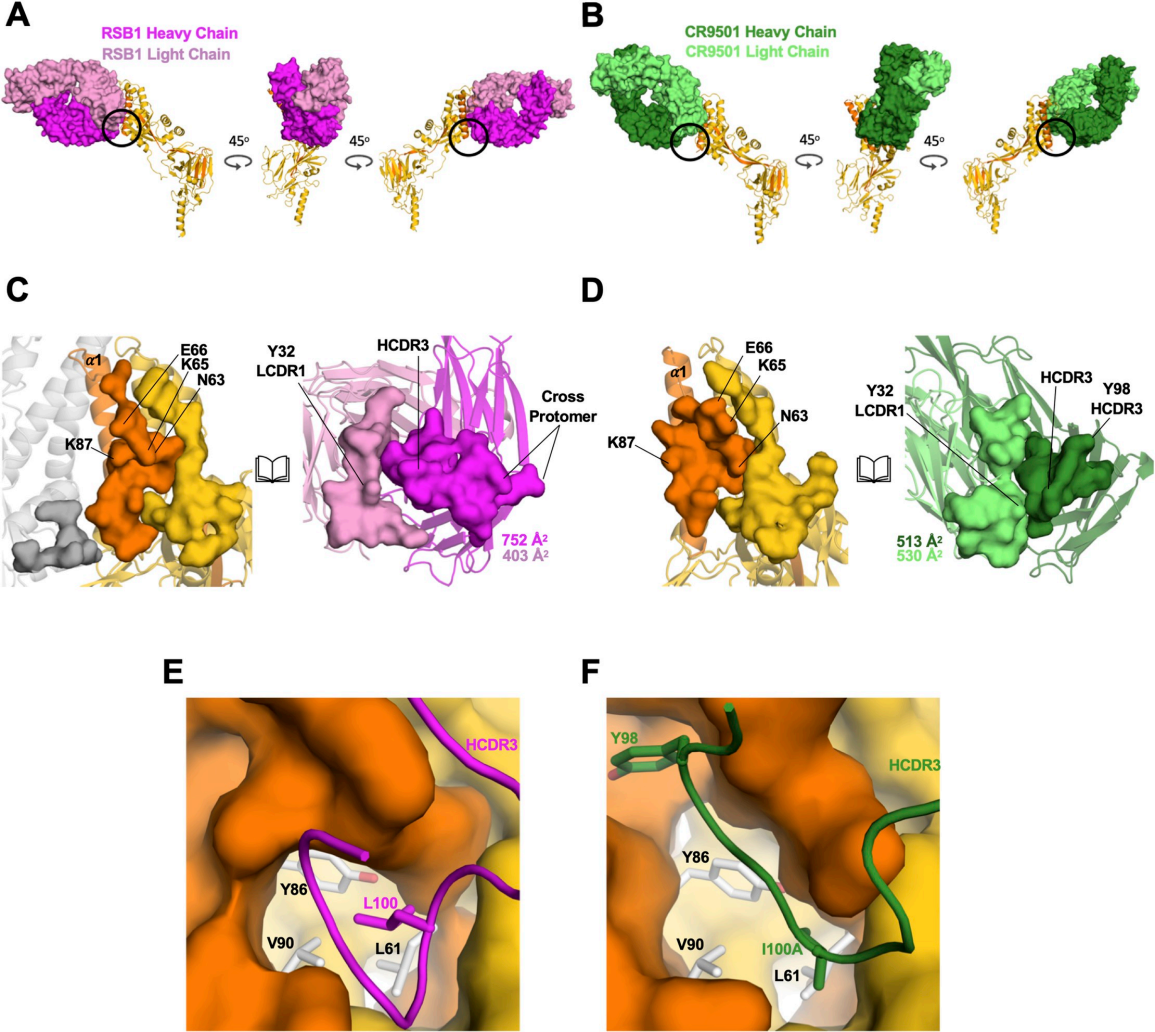
RSB1 and CR9501 target similar epitopes with different angles of approach and an “induced fit”. Three views, each rotated by 45 degrees for **A**) DS-Cav1-RSB1 complex and **B)** CR9501 complex. For simplicity, only one protomer is shown bound by one Fab. CR9501 is colored dark green for the heavy chain and light green for the light chain. RSB1 coloring matches Fig 1. **C-D**) Open-book views of the RSB1 (C) and CR9501 (D) interfaces showing total buried surface areas for their respective epitopes and paratopes. **E-F)** HCDR3 for RSB1 (E) and CR9501 (F) are shown as cartoons, while F1 and F2 are depicted as surface. Residues Leu100 (RSB1) and Ile100A (CR9501) are shown with sticks. Tyr98 on CR9501 which causes an induced fit is also shown as sticks.

RSB1 is the third site V-directed nAb whose structure is being reported bound to a prefusion stabilized F. The nAb hRSV90 (PDB 5TPN) was solved bound to SC-TM PreF (N67I, S215P and E487Q), while nAb CR9501 (PDB 6OE4) was reported bound to a monomeric form of DS-Cav1[33, 37]. The angle of approach to the epitopes targeted by hRSV90 and RSB1 differ by approximately 60^o^ relative to the perpendicular axis through PreF, with each antibody recognizing opposite sides of the α3 helix (**S8A Fig**). Thus, while both are classified as binding to antigenic site V, there is minimal overlap between the two epitopes. Conversely, though originating from diverse germlines, RSB1 (V_H_1-69) and CR9501 (V_H_4-31) recognize the PreF molecule in a remarkably similar manner, with significant overlap between their respective binding sites (**Fig 5A–5B and S8B Fig)**. Interestingly, CR9501 was shown to favor the opening of soluble, prefusion F trimers, and the crystal structure in ternary complex with Motavizumab (PDB 6OE5) indicated that CR9501 was bound to a single protomer of a “splayed open” PreF trimer [37]. By contrast, our biochemical and structural analysis of RSB1 shows no indication for opening of the DS-Cav1 trimer, as also confirmed by the DS-Cav1-RSB1 complex crystallizing in the trimeric state. Despite these different oligomeric preferences, CR9501 also shows high neutralization potency against RSV A and B strains, with IC_50_ values of ~9–35 ng/mL [37], comparable to that of RSB1 reported here. RSB1 and CR9501 each bury an extensive surface with their heavy and light chains (**Fig 5C and 5D**); however, due to a rotation of the RSB1 variable domain by 21^o^ relative to CR9501, RSB1 buries a portion of the adjacent protomer, as described earlier. These RSB1 specific cross-protomeric interactions may serve to stabilize the PreF trimer and prevent it from transitioning to the open state observed for CR9501 binding.

**Fig 6:**
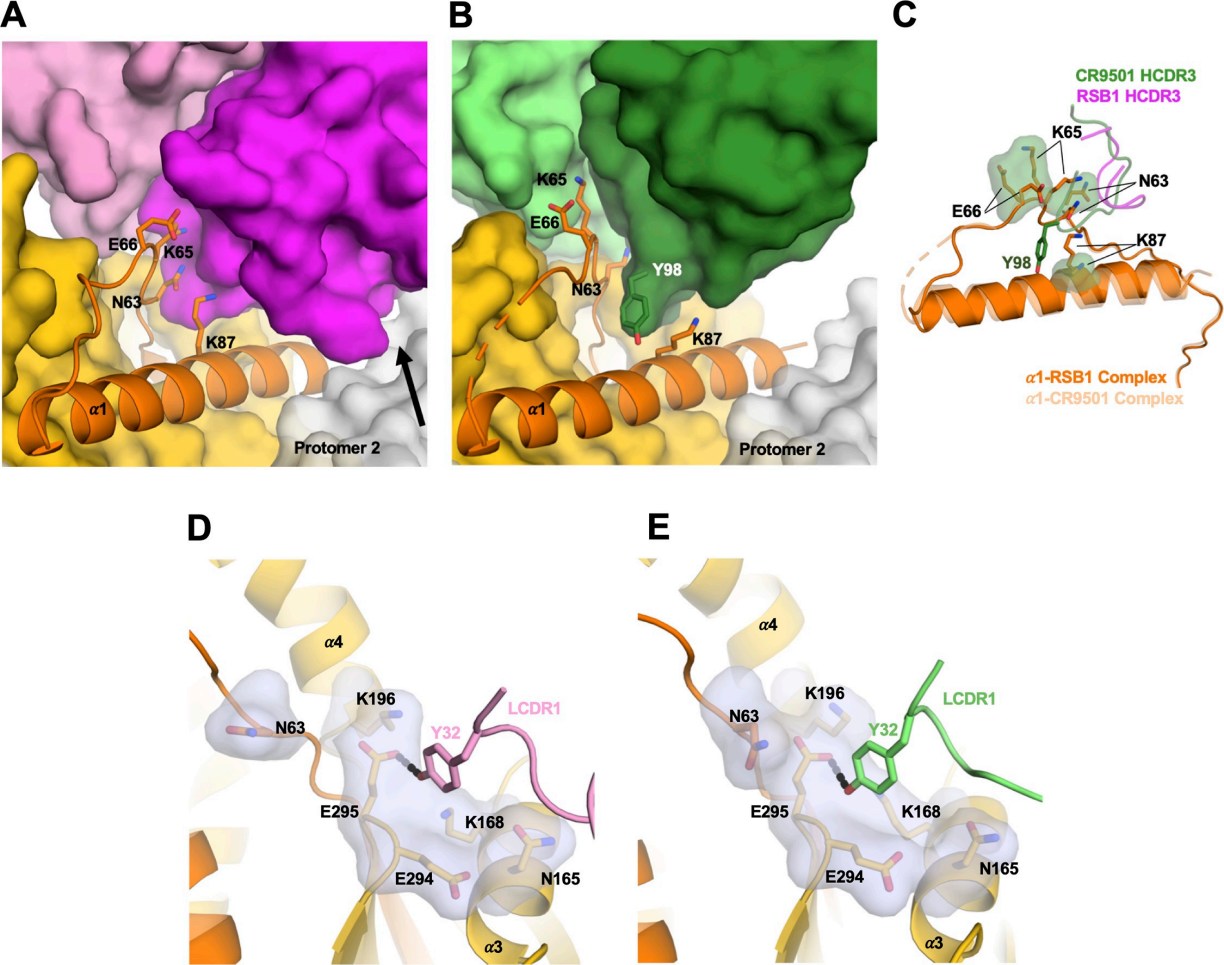
Structural comparison between DS-Cav1-RSB1 and CR9501 complexes reveals an induced fit and convergent structural features. **A-B)** View down the membrane distal apex of the prefusion F molecule for RSB1 (A) and CR9501(B) complexes. The α1 helix is shown in cartoon and colored orange. Protomer 2 is colored white. Residues on PreF that shift upon binding of CR9501, due to an “induced fit” caused by CR9501 Tyr98 are shown as sticks. The black arrow shows where RSB1 forms cross protomer interactions that are not found in the CR9501 complex. **C)** Superposition of the RSB1 and CR9501 complexes showing a zoomed-in view of the “induced fit”. For clarity, the helix for the CR9501 bound complex is shown as transparent cartoon. Residues shifted when CR9501 binds are shown as sticks with a green transparent surface, whereas the corresponding residues on the RSB1 complex are shown as sticks with no surface. The HCDR3 is shown as cartoon for each Fab, with the CR9501 Tyr98 shown as a stick. (**D-E**) Zoomed view of the LCDR1 Tyr32 interaction with F1 for RSB1 (D) and CR9501 (E).

Despite variation in the angle of approach towards PreF, RSB1 and CR9501 each insert HCDR3 into the same hydrophobic pocket. RSB1, utilizing Leu100_HCDR3_, and CR9501 utilizing Ile100A_HCDR3_, each make van der Waals contacts with F2 residues Leu61, Tyr86, and Val90 within this hydrophobic patch (**Fig 5E and 5F**). The HCDR3 of CR9501 is 15 residues in length, compared to twelve residues of RSB1, and the longer HCDR3 allows CR9501 to position Tyr98_HCDR3_ into a second pocket at the center of helix α1 (**Fig 5F**). Interestingly, this pocket is not accessible on the DS-Cav1-RSB1 complex, and thus likely results from an “induced fit” caused by the displacement of F2 residues Lys87 and Glu66, which are each shifted by ~4 Å between the two complexes (**Fig 6A–6C**). Additionally, F2 residues Asn63 and Lys65 are also shifted between the two structures by ~4 and ~8 Å, respectively. In the case of Lys65, this movement allows it to be positioned at the interface of the heavy and light chains for either complex (**Fig 6C**). As shown earlier in our mutational analysis, these two particular residues contribute significantly to the binding of RSB1, so it is possible that the flexibility of these side chains is a key component to the antigenicity of this epitope.

### Convergent structural features between RSB1 and CR9501

For light chain interactions, RSB1 utilizes the IGLV2-11 germline gene, whereas CR9501 uses IGKV1-33. Interestingly, each antibody contains a Tyr32_LCDR1_ and comparison of the two complexes reveals that this tyrosine adopts nearly identical spatial orientations, inserting into a groove between α3 and the loop connecting β5-β6, forming a hydrogen bond with F1 residue Glu295 (**Fig 6D and 6E**). Helix α3 is one of the structural elements of the heptad repeat A (HRA) region that rearranges to form the center of the six-helix bundle in PostF [41]. Tyrosine has the highest propensity to be located at antibody-antigen interfaces and is preferred for interactions with Asp and Glu [42]. Thus, this particular interaction, which is shared between RSB1 and CR9501 despite originating from separate germlines, may be a key, evolutionarily preferred determinant for recognizing the prefusion F conformation.

The use of a Tyr32_LCDR1_ for each of the complexes led us to investigate the frequency for this residue in sequences of antibodies targeting site V, as reported by Gilman et al. [30]. From the forty-five sequences available, there are five distinct light chain germlines, representing 86% of the total available sequences, which are all predicted to have a germline encoded Tyr32_LCDR1_ (**S9A Fig**). Combined with the RSB1 and CR9501 sequences, this resulted in six unique light chain germlines (as mAb ADI-18977 shares the same light chain as CR9501) and ten unique heavy and light chain germline pairs. Each of the LCDR1 regions were of different lengths, therefore we sought to ensure that the sequence alignment corresponded to the expected 3D shape. Therefore, we generated homology models for one representative light chain from each unique germline using the Swiss Model server [43] and compared these with RSB1 and CR9501. Alignment of the structures confirmed that the LCDR1 tyrosine for each antibody assumes similar positioning as in the RSB1 and CR9501 complexes (**S9B Fig**). Additionally, each of these antibodies contains either a small hydrophobic or polar uncharged residue (valine, alanine, serine, threonine, or glycine) near the tip of HCDR3, which we hypothesize could be accommodated in the same groove occupied by the RSB1 and CR9501 HCDR3 residues (Leu100 and Ile100A, respectively). Remarkably, these analyses also suggest that a large portion of the antibody repertoire targeting site V may be binding the RSB1/CR9501 epitope.

### Computational energetics support role for Tyr32LCDR1 in binding of RSB1 and CR9501

**Fig 7:**
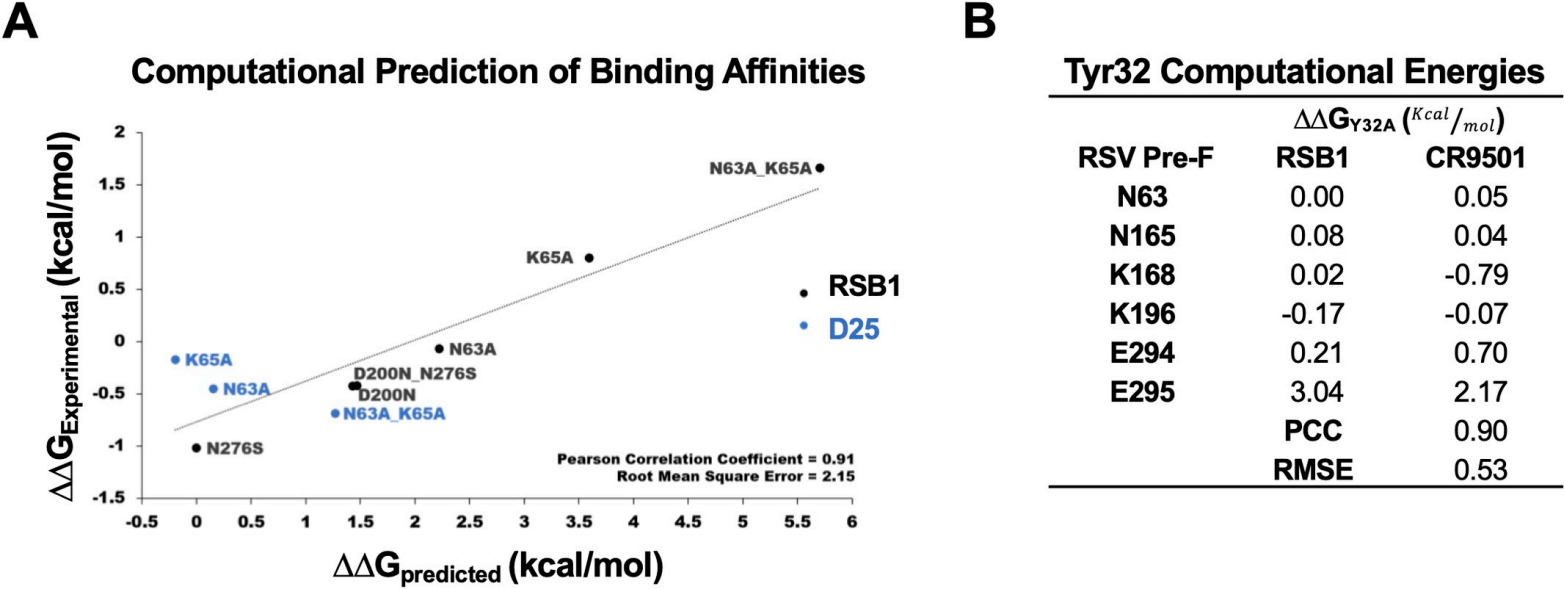
Computational analysis of RSB1 binding. **A)** Calibration of Rosetta *in silico* computations by comparison of experimental data with *in silico* binding affinity calculations for RSB1 and D25 alanine mutations, and RSB1 RSV B substitutions. Mutation free energy is calculated as the change in Gibbs Free Energy (ΔΔG) between the mutant and wildtype residues. **B)** Difference in pairwise free energy (kcal/mol) for an *in silico* Tyr32Ala mutation in the RSB1 and CR9501 LCDR1. **PCC:** Pearson’s Correlation Coefficient **RMSE:** Root Mean Square Error.

To explore the significance of Tyr32 for recognition and binding to PreF, we used *in silico* binding affinity prediction tools from the Rosetta Protein Design Suite to calculate interface energies from computational Tyr32Ala mutations in the RSB1 and CR9501 complexes. In order to first calibrate the accuracy for these calculations, as well as gain additional energetic details about the DS-Cav1-RSB1 complex, we utilized Rosetta to make *in silico* mutations based on the experimental data we had generated using our alanine and RSV B mutants. The computationally-derived interface energies were compared to experimental data by converting *in vitro* K_D_ values measured by SPR to the relative Gibbs free energy between wildtype and mutant residues, or ΔΔG in kcal/mol, as described previously [44–46] (**Fig 7A**). This comparison between simulation and experiment indicated that a threshold of ± 2 kcal/mol was necessary to describe stabilizing energetics (ΔΔG or Gibbs free energy < -2 kcal/mol) or destabilizing mutations (ΔΔG > 2 kcal/mol). Importantly, we confirmed that there is high correlation between the experimental and *in silico* predictions, thus lending confidence towards our calculations applied to LCDR1 Tyr32Ala mutations (S1 Data).

As anticipated, the *in silico* Tyr32Ala mutation predicted a large loss in binding affinity for both the RSB1 and CR9501 complexes, with similar ΔΔG values of 2.9 and 3.1 kcal/mol, respectively, thus suggesting considerable interface destabilization. Analysis of individual epitope contributions indicated that the affinity of each antibody for PreF is primarily driven by the hydrogen bond between Tyr32 and Glu295 (RSB1: ~3 kcal/mol, CR9501: ~2.2 kcal/mol); however, adjacent residues also add minor contributions to the binding energy (Lys168 and Glu294 on CR9501; Lys196 and Glu294 on RSB1) (**Figs 6D, 6E and 7B**). This highly correlated network of interactions seems to explain the requirement for the tyrosine in this location, which as described earlier, resides at a junction that rearranges in PostF. Thus, an abundance of light chain germlines which encode this necessary residue, and their ability to pair with multiple heavy chains which present the necessary hydrophobic HCDR3 residue(s), may explain the apparent preference for this particular site V epitope.

### Immunization with DS-Cav1 elicits RSB1-like antibodies

**Fig 8:**
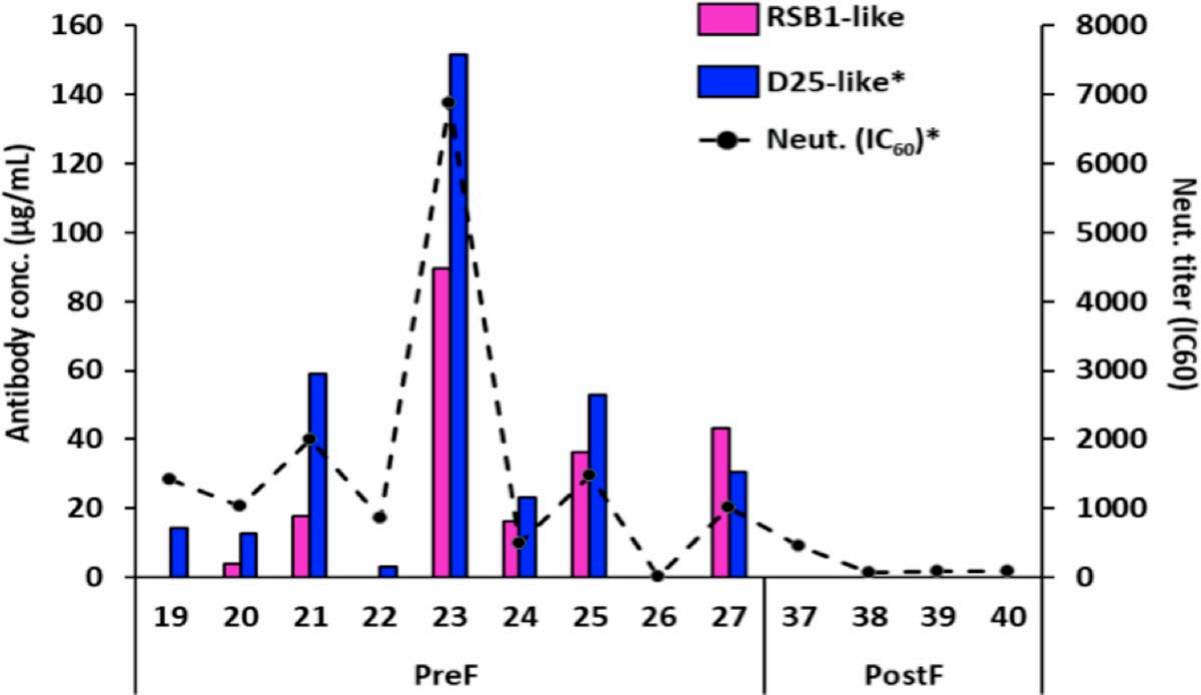
RSB1-like antibodies are induced upon immunization with DS-Cav1. **A)** In a naturally-primed bovine model, RSB1-competing antibodies trend similar to D25-competing antibodies. RSV A neutralization titers are represented by the broken black line. The PostF antigen did not induce measurable neutralizing, or RSB1- or D25-competing antibodies in this study. *The values for D25-like antibodies and RSV A neutralization titers were previously reported in Steff *et al*., Nature Communications (2017) [17].

We have previously reported on the ability of DS-Cav1 to induce a high neutralizing antibody response in a naturally-primed bovine model [17]. Animals immunized with DS-Cav1, but not a PostF antigen, showed a measurable increase in D25-like antibodies following a single immunization. We re-tested the serum samples from that study to understand if a similar trend could be observed for RSB1-competing antibodies. The results confirmed that RSB1-like antibodies were indeed induced following immunization with DS-Cav1, but not PostF (**Fig 8**). Furthermore, the trend closely correlated with that of D25-like antibodies, and further correlated with the overall neutralizing titers in these sera. These observations suggest that the RSB1 epitope on DS-Cav1 is robustly immunogenic, and binding antibodies are induced or boosted above detection following immunization. The highly similar trend between these antibody levels and the neutralizing titer in the sera further suggest that antibodies to this site contribute to the overall neutralizing response raised by this antigen.

## Discussion

The fusion protein F is indispensable for the RSV life cycle and is highly conserved across different subtypes of the virus. Furthermore, the prefusion conformation of F is the predominant target of neutralizing antibodies (nAbs) induced by natural infection, making it the leading candidate antigen for vaccine development. Compared to other forms of F, stabilized prefusion F elicits high levels of nAbs not only in naïve and naturally-primed animal models [17, 22, 47], but also in healthy adults, as evidenced by recently published data from phase 1 clinical trials [24]. This has led to a growing interest in understanding the protective epitopes on the prefusion F surface, and the antibodies targeting these regions of susceptibility. The discovery, isolation, and structural characterization of nAbs targeting PreF has thus far elucidated multiple antigenic sites on its surface, as well as provided clarity on RSV subtype preference, inherent flexibility and breathing of the PreF trimer [22, 28, 32, 33, 37, 38].

Here, we report the structure and properties of prefusion stabilized F (DS-Cav1) bound by RSB1, an RSV neutralizing human monoclonal antibody that specifically recognizes the prefusion conformation of F. Although RSB1 was originally identified as a competitor to the site Ø binding mAb D25, epitope mapping based on the crystal structure and HDX-MS reveal that it in fact binds antigenic site V, which only marginally overlaps with site Ø. The structural and mutational studies reveal how the strong competition with D25 most likely arises from steric clashes between the Fab regions of the antibodies, due to both mAbs recognizing minimally overlapping epitopes. Additionally, the RSB1 structure elucidates cross-protomeric interactions which overlap with a portion of the Motavizumab epitope, explaining this competition. The RSB1 epitope is comprised of residues that are highly conserved across RSV A and B strains, in agreement with the high neutralization potency of RSB1 against both viruses. The high specificity of RSB1 for PreF, and the extensive rearrangement of the residues within its epitope when F adopts the PostF conformation, suggest that RSB1 potentially neutralizes RSV by trapping F in its prefusion conformation, thereby impeding its fusogenic function required for viral entry into host cells.

Prior to this study, the epitopes of two other monoclonal antibodies that target site V, hRSV90 and CR9501, had been structurally characterized. Comparative analyses of the three structures reveal that hRSV90 binds exclusively to F1 on alpha helices α3, α4, and beta sheets β3–4, while RSB1 and CR9501 bind to PreF on the opposite face of helix α3 in a remarkably similar manner. Unlike hRSV90, the latter two also exhibit extensive contacts on F2, and share a novel structural signature in their HCDR3 and LCDR1. With the solving of the RSB1 structure, such similarities and potential structural requirements for antibodies targeting antigenic site V can now be comprehensively defined.

Comparison of the RSB1 and CR9501 binding modes suggests how convergent structural features, such as a hydrophobic HCDR3, and a germline encoded Tyr32 on LCDR1 may be important elements for recognition of this PreF epitope. Tyrosines targeting a specific location of a viral protein has been seen in other pathogens such as influenza, where a conserved tyrosine in the HCDR3 of V_H_1-69 derived antibodies recognizes a pocket on the hemagglutinin stem [48]. This same interaction is mirrored with other heavy chain germlines, such as 16.a.26 which utilizes the HCDR2 of V_H_1-18[49]. In the case of influenza, the tyrosine interaction can also be contributed by the light chain, as seen with mAb MEDI8852 [50]. Future structural studies of antibodies targeting site V of RSV PreF are needed to advance our understanding of the binding requirements outlined here. Nonetheless, sequencing of B cells post vaccination and searches for this signature might allow a more rapid means for predicting and quantifying the contribution of this epitope to the immune response. Additionally, plasticity of this site V epitope, shown through an induced fit of the CR9501 HCDR3, as well as the flexible lysine and asparagine residues, may allow for a larger selection of nAbs to bind, consistent with RSB1 and CR9501 originating from distinct germlines.

Despite their similarities, RSB1 and CR9501 also differ dramatically in their preference for the oligomeric state of PreF. While RSB1 binds to trimeric PreF, and even harbors cross-protomer interactions, CR9501 preferentially binds to a more open PreF conformation once the trimeric PreF head is splayed open, or monomeric PreF [37]. This difference is unlikely to arise from the specific contacts spread across F1 and F2, a feature shared with RSB1. A more plausible explanation is that the induced fit of the PreF surface residues upon binding of CR9501, especially its long HCDR3, may trigger an allosteric effect destabilizing the inter-protomer interface. Conversely, the cross-protomer contacts of RSB1 HCDR1 on the PreF surface may have a stabilizing effect on preserving its trimeric conformation when bound by this antibody. Although each antibody prefers binding a different oligomeric state of PreF, each is also able to neutralize RSV A and B strains with similarly high potency. This may suggest that the shared mechanism of neutralization (effective trapping of F in the prefusion conformation) is more important than monomer or trimer specificity. The ability of the immune system to target the structural dynamics of the RSV F protein, inherent of a monomer-trimer equilibrium, appears to allow antibodies with differing oligomeric preferences to recognize and efficiently neutralize the virus.

The finding that diverse germlines can give rise to antibodies with convergent structural features targeting antigenic site V is in contrast to the known anti-RSV antibodies targeting other antigenic sites. Comparatively, three different human antibodies have been structurally characterized bound to site Ø (D25, AM22 and RSD5); however, there is no apparent common structural motif within the antibody CDRs. One implication of such convergent features could be that a diverse, naturally-primed human population would be readily capable of mounting an immune response to antigenic site V in response to PreF-based vaccination. As a first clue, we observed that in naturally-primed cattle, PreF was able to robustly boost an immune response to site V. As the RSV field progresses in clinical development, it will be interesting and informative to understand how site V immunogenicity contributes in the human setting, and the deep characterization of molecular tools such as nAb RSB1 will further aid the process of vaccine development.

## Methods

### RSV A and B neutralization assay

Monoclonal antibodies (mAbs) were serially diluted 2-fold in medium containing DMEM, 3% FBS, 1μg/mL gentamicin and 2mM glutamine (RSV medium). Eight serial dilutions were carried out in a 96 well U bottom plate for each mAb in two experiments. The antibodies were tested at two starting concentrations, 0.1 μg/mL and 2μg/mL. Cotton rat serum was used as positive control. MAbs and positive control serum dilutions were mixed with RSV A Long and RSV B 18537 (ATCC), diluted to approximately 100 pfu/well and were incubated for 2 hours at 35°C. After incubation, the virus-antibody/serum mixture was transferred to 96-well plates previously seeded with Vero cells after removing the growth media from the plates. On each plate, 16 wells were control wells incubated with virus only (100% infectivity). Plates were incubated for 2 hours at 35°C, virus-antibody/serum mixture was removed from the cells and RSV medium containing 0.5% Carboxymethylcellulose (medium viscosity) was added to all wells. The plates were incubated for 42–45 hours at 35°C before staining.

Cell monolayers were fixed with 10% neutral buffered formalin for 60–70 minutes. After fixing, the cells were washed with PBS/0.05% Tween and blocked with DPBS/0.5% saponin/2.5% FBS/0.1% sodium azide for an hour. The blocking buffer was removed, and RSV-positive cells were detected using an RSV fusion protein mAb (1:1000 dilution of antibody in blocking buffer) (Bio-Rad) and an RSV nucleoprotein mAb (1:1000 dilution of antibody in blocking buffer) (Bio-Rad) and incubated for 1 hour at room temperature. The primary antibody was washed with PBS/0.05% Tween from the plates and cells were stained using goat F(ab’)_2_ conjugated to HRP (1:1000 dilution of antibody in blocking buffer) (Southern biotech) and incubated for 1 hour at room temperature. After washing the secondary antibody with PBS/0.05% Tween from the plates, True Blue substrate was added to all wells for 10–15 minutes and the plates were washed with distilled water. The plates were allowed to air dry in the dark for 10–15 minutes. Plates were scanned and counted on the Cellular Technologies Limited-ELISpot reader. Plaque counts of mAbs tested at the 0.1 μg concentration were selected to calculate inhibition curves. IC_60_ values for mAbs were calculated by extrapolating the inhibition curves using Graphpad Prism version 8.

### Expression and purification of DS-Cav1 and RSB1 variants

DS-Cav1 produced in CHO cells was purified by affinity and ion exchange chromatography. PreF mutants were cloned on DS-Cav1 with a C-terminal thrombin-cleavable double Strep tag II followed by a His-tag as a template. Single or multiple point mutations were generated using the Q5 Site-Directed Mutagenesis Kit (New England Biolabs, Ipswich, MA). Proteins were transiently expressed in Expi293 cells (Thermo Fisher Scientific, Carlsbad, CA), and purified using affinity chromatography followed by removal of Strep/His tags using Thrombin protease, and a final size exclusion chromatography polishing step, as previously described [17].

RSB1 wildtype and mutant IgG was recombinantly expressed in Expi293 cells and purified using Protein A and size exclusion chromatography, as previously described [17]. Mutations in RSB1 sequence were generated using site directed mutagenesis using the Q5 Site-Directed Mutagenesis Kit (New England Biolabs, Ipswich, MA). RSB1 Fab was expressed with a Strep Tag II at the heavy chain C terminus and purified using a StrepTrap HP column (GE Healthcare). The tag was proteolytically cleaved using TEV protease (AcTEV protease, Thermo Fisher Scientific) prior to size exclusion chromatography. DS-Cav1 was incubated with a 1:1.5 molar excess of RSB1 Fab prior to size exclusion chromatography to prepare the complex for crystallization.

### Crystallization and X-ray data collection

Apo RSB1 Fab protein was concentrated to 10mg/mL in buffer containing 10mM Hepes pH 7.5, 150mM NaCl, and 5% glycerol. Crystals formed by hanging drop vapor diffusion at a 1:1 ratio of protein to crystallization buffer containing 0.2M ammonium sulfate, 0.1M bis-tris pH 5.5, 25% w/v PEG 3350. DS-Cav1 bound to RSB1 Fab was concentrated to 6mg/mL in buffer containing 10mM Hepes pH 7.5, 150mM NaCl, and 5% glycerol. Protein crystals for DS-Cav1 bound to RSB1 Fab were identified in buffer containing 0.1M Hepes pH 7.5, 25% PEG 2000 MME. An additive screen (Hampton Research) was set up and more than a dozen additives resulted in crystal growth of similar morphology, but in some cases larger, and/or visibly sharper edges were observed. Crystals for Apo RSB1 and DS-Cav1-RSB1 complex were cryo-protected with their respective mother liquor supplemented with 10% ethylene glycol and shipped to the Advanced Photon Source (APS) at Argonne National Labs for X-ray data collection. Crystals of Apo RSB1 diffracted to 2.0 Å, while crystals of DS-Cav1-RSB1 obtained with the additive spermidine diffracted to a resolution of 3.7 Å. X-ray diffraction data were collected and scaled in *P* 2_1_ and *I* 2_1_3 space-groups for the RSB1 Apo and DS-Cav1 bound structures, respectively.

### Structure determination, model building, and refinement

Diffraction data were indexed and scaled with HKL2000[51], and Phaser [52] was used for molecular replacement using PDB 4MMU as a search model for DS-Cav1, and a homology model of RSB1 built using the PIGS server (Prediction of ImmunoGlobulin Structure)[53] as the second search template. Iterative rounds of reciprocal space and real space refinement were carried out in Phenix [54] and Coot [55]. The final structures were refined to *R*_work_/*R*_free_ values of 22/29% for both, the DS-Cav1-RSB1 complex and Apo RSB1 (**S1 Table**). The asymmetric unit of the complex structure is made of one protomer of the DS-Cav1 molecule and one RSB1 Fab, and the trimeric DS-Cav1 with three RSB1 Fabs bound was reconstituted by applying symmetry operators. Final coordinates were deposited in the Protein Data Bank with accession codes 6W5D and 6W52 for the RSB1 Fab and DS-Cav1-RSB1 Fab complex, respectively. Structure figures were made using PyMOL [56] and ChimeraX [57].

### Surface plasmon resonance experiments

Single cycle kinetics experiments were performed in triplicate on a Biacore T200 using a ligand capture method at 25°C. A CM5 chip was immobilized with human Fc binder using the Human Antibody Capture Kit, according to manufacturer’s recommendations. HBS-EP buffer was used as both running buffer and sample diluent. IgGs were captured to 100–200 RUs in one flow cell, leaving the other as reference. DS-Cav1 and its variants were injected into both flow cells at 50μL/min for 120s followed by 600s dissociation. Antigen concentration ranged from 0 – 20nM, and reference- and blank-subtracted sensograms were fitted using a 1:1 binding model to calculate k_on_, k_off_ and K_D_.

### Biolayer interferometry competition assay

Antibody competition assay on DS-Cav1 was performed using the Octet Red instrument (FortéBio Corp). 6xHIS-tagged DS-Cav1 and mAbs were diluted in 1× PBS with 1% BSA at the final concentration of 20 μg/ml and assay was run at 30°C. 6x-His-tagged DS-Cav1 was captured onto His biosensors for 30 s in 1× PBS with 1% BSA. Typical capture levels were between 1.2 and 1.4 nm. Biosensor tips were washed for 30 s in 1× PBS with 1% BSA before capturing primary and secondary mAbs respectively. Binding of primary and secondary mAbs was measured for 300 s. Binding inhibition was calculated by the following equation: inhibition (%) = 100 − (secondary mAb binding in the presence of primary mAb) / (secondary mAb binding in absence of primary mAb) × 100. The values reported are the average of three independent experiments, plus or minus standard deviation.

### HDX-MS

The DS-Cav1-RSB1 Fab complex was formed by mixing 17.2 μM of DS-Cav1 with 31.33 μM of RSB1 Fab, for approximately a 1:2 antigen:antibody ratio. HDX-MS analysis was performed on a Waters HDX manager with a LEAP system coupled to a Xevo G2-S QTOF mass spectrometer. All test materials were analyzed in triplicate. Each sample, DS-Cav1 or DS-Cav1-RSB1 complex, was diluted 20-fold in 10 mM potassium phosphate in D_2_O, pD 7.0 and incubated for 0.5, 1, 5, 10, 30, and 120 minutes. Hydrogen-deuterium exchange was quenched at 0°C with equal volume of 10 mM potassium phosphate,1 M Guanidine hydrochloride, 0.25 M Tris (2-carboxyethyl) phosphine hydrochloride (TCEP) at pH 2.5. Samples were immediately digested on an on-line enzymate pepsin column (Waters) at 20°C. Peptic peptides were eluted in 4 minutes with 0.1% formic acid in water at a flow rate of 100 μL/min and concentrated and buffer exchanged on Waters Vanguard C18 pre-column (2.1x50 mm), and then separated with a 5–35% gradient of 0.1% formic acid in acetonitrile over 7 minutes at a flow rate of 40 μL/min on Acquity BEH C18 column (1.7 μm, 1.0 X 100 mm, Waters) at 0°C. The peptic peptides were identified using collision induced dissociation (CID) MS/MS performed with data independent MSE acquisition. Mass spectra were processed using ProteinLynx Global Server (PLGS) and deuterium incorporation for each peptide was quantified using DynamX software (version 3.0.0) from the centroid mass difference between deuterated and non-deuterated samples.

### Computational binding free energy of RSB1 and RSV PreF mutants

#### Minimization

The initial 3.7 Å structure of the DS-Cav1-RSB1 complex was used as a template for the prediction of binding affinity calculations. Two rounds of minimization in the Rosetta Protein Design Suite [44] were required for convergence, utilizing the Rosetta Fast Relax algorithm and the beta_cart force field, which generated twenty models from which a minimum energy structure was obtained. The 3.6 Å PreF-D25 complex (PDB 4JHW) was initially minimized with the YASARA Structure molecular dynamics package and the YASARA2 force field [58], due to poor convergence of the crystal structure during the Rosetta relaxation steps. This MD-based minimization was followed by two rounds of relaxation in Rosetta, resulting in twenty models from which the minimum energy pose was chosen. The 3.3 Å DS-Cav1-CR9501 complex (PDB 6OE4) was used as the starting structure for the calculations to analyze the preservation of the Tyr32 residue contacts between RSB1 or CR9501 and PreF. Also, in this case, two rounds of minimization using Rosetta Fast Relax and the beta_cart force field were necessary to achieve convergence in the Rosetta energy score.

#### Mutagenesis & energy calculations

The Cartesian ΔΔG module in the Rosetta Protein Design Suite [46] was used on the minimized structures to generate the point mutations of interest, mutating to both the wildtype and target residue, flexibly minimizing neighboring residues within a 6Å spherical window, and generating 100 structures for both wildtype and mutant. The Protein Interfaces, Surfaces and Assemblies software, PISA, obtained from the European Bioinformatics Institute [59] was then used to identify the interface residues between antibody and antigen, as input for interface energy calculations with Rosetta [45]. The average interface energy from the 100 models was analyzed via Pearson’s correlation coefficient (to calculate level of association) and Standard Error (to estimate deviation) against experimental free energies converted from SPR data [60]. Equations for the calculations are shown below; where R is equal to the Universal Gas Constant, 1.987 x 10–3 kcal∙K^-1^∙mol^-1^, T is equal to 298.15 Kelvin, K_D_ is the experimental k_d_/k_a_ from the SPR data, PCC is the Pearson’s correlation coefficient, and RMSE is the Root Mean Square Error:

$$\Delta G={\mathrm{RTlnK}}_{D}\mathrm{PCC}=\frac{\mathrm{covariance}(\Delta \Delta G_{\mathrm{experiment}},\Delta \Delta G_{\mathrm{predicted}})}{{\mathrm{variance}}_{\Delta \Delta G_{\mathrm{expt}}}\cdot {\mathrm{variance}}_{\Delta \Delta G_{\mathrm{pred}}}}\mathrm{RMSE}=\sqrt{\frac{\sum_{1}^{n}{\left(\Delta \Delta G_{\mathrm{expt}}‐\Delta \Delta G_{\mathrm{pred}}\right)}^{2}}{n}}$$

### RSB1 competition assay

A competition ELISA was performed to determine the concentration of RSB1-like antibodies in serum. RSB1 antibody (referred to as “tracer”) was biotin conjugated using a commercial kit according to manufacturer instructions (EZ-Link NHS-PEG4-Biotin, No-Weigh Format, Thermo Scientific). 96-well ELISA plates (Immuno F96 MaxiSorp, Nunc) were coated with 100 μl/well of DS-Cav1 diluted to 2 μg/ml in Phosphate Buffered Saline (PBS). Following overnight incubation at 4°C, wells were washed with PBS containing 0.05% (w/v) Tween 20 (wash buffer). The wells were blocked with 1% (w/v) bovine serum albumin (BSA) in PBS for 90 min at room temperature (RT). Bovine serum samples (starting 1:10) or unlabeled RSB1 (standard, starting at 8 μg/ml) were serially diluted two-fold in PBS containing 1% (w/v) BSA and 0.1% (w/v) Triton X-100 (sample buffer). Sample and standard dilutions were combined in equal volumes with 30 ng/mL tracer. The ELISA plates were washed and tracer-sample/standard mixtures were transferred to the plates. Eight wells contained tracer only for determination of the tracer signal (tracer-only binding). Plates were then washed and incubated with HRP-conjugated Streptavidin (Vector cat# SA-5004) diluted 1:5000 in sample buffer at 100 μl/well for 1 h at RT, followed by a wash and incubation for 20 min with 100 μl/well of TMB substrate (Rockland cat# TMBE-1000) at RT. Following incubation, the reaction was stopped by adding 100 μl/well of 2.0 N Sulfuric Acid (BDH cat# BDH3500). The optical density was determined at 450 nm using a microplate reader (Molecular Devices iD3).

Percent inhibition of the tracer-only binding was calculated for each standard and sample dilution and plotted according to concentration (standards) or dilution (samples). For standards, the concentration of unlabeled RSB1 leading to 50% inhibition of the corresponding tracer (EC50) was calculated in Softmax Pro. For samples, the dilution corresponding to 50% inhibition was calculated in a similar manner, but only if the lowest sample dilution (1:10) was above 50% inhibition. This dilution was multiplied by the RSB1 (standard) EC50 concentration to establish RSB1-like concentrations in each sample.

## Supporting information

S1 Fig Epitope mapping using HDx-MS. **(A)** Deuterium uptake plots for four peptic fragments of DS-Cav1 alone (blue) and for the complex of DS-Cav1 and RSB1 (red) showing peptides with different deuterium uptake upon complex formation. (B) HDx-MS fragments mapped onto PreF structure. Framgents starting at residues 93 and 199 correspond to portions of site Ø, while fragments starting with residues 57 and 161 correspond to site V.
(PDF)

S2 Fig **A)** 2Fo-Fc electron densities (contoured at 1 sigma) are shown as mesh around one protomer of DS-Cav1 in complex with one RSB1 Fab, as well as zoomed at the heavy chain interface with DS-Cav1. **B)** 1σ 2Fo-Fc electron densities around the CDRs for the RSB1 Apo structure.
(PDF)

S3 Fig RSB1 epitope residues defined by X-ray crystallography are clustered on the PreF structure (left, cyan patches). In contrast, in the PostF structure (right), the same residues (cyan) are rearranged in a much more dispersed manner, indicated by black arrows. Distances indicate approximate movement from residue locations in PreF structure.
(PDF)

S4 Fig RSB1 heavy (magenta) and light (pink) CDRs are shown with cartoon and side chain sticks, while the RSB1 epitope on PreF is shown as surface and colored according to the residue conservation between RSV A and B strains generated using the ConSurf server [61]. Conservation is indicated by a gradient from dark red (highly conserved) to green (not conserved). Residues in the epitope which are not conserved between A and B strains (D200, N276) are labeled in green.
(PDF)

S5 Fig Structural basis for competition with Mota. The RSB1 epitope is colored in cyan. Mota heavy chain is colored dark brown and light chain is colored light brown. The Mota Fab bound to the same PreF protomer as a single RSB1 Fab is labeled as Protomer 1, whereas the Mota Fab that competes with RSB1 binding is labeled as the adjacent protomer. The third Mota Fab is not visible from this view. The RSB1 cross-protomer contacts which overlap with the Mota epitope are labeled.
(PDF)

S6 Fig Difference in surface potential for PreF residue Asp200/Asn200 in RSV A and B viruses. **A)** Surface potential around PreF residue Asp200 from this study (left), representing RSV A, and the surface potential on RSB1 around the Arg53_(LCDR2)_ which interacts with Asp200 (right). **B)** Surface potential around PreF residue Asn200 from the structure of RSV B PreF PDB 6Q06 (left), with the RSB1 interacting residue Arg53_(LCDR2)_ is shown again for comparison (right).
(PDF)

S7 Fig RSB1 maintains global conformation between bound and unbound states. **A)** Comparison of the variable fragments for the RSB1 apo structure (colored white) and the DS-Cav1-RSB1 bound structure (colored as previously). **B)** Zoomed view of Tyr29_HCDR1_ which is re-orientated with respect to the unbound state. Interactions with three residues across two DS-Cav1 protomers are shown as sticks with transparent surface. **C)** Zoomed view of Arg53_LCDR2_ which is also re-orientated with respect to the unbound state. Salt bridge is shown for interaction with F1 residue Asp200, which is substituted with Asn200 in RSV B viruses.
(PDF)

S8 Fig Two distinct epitopes at antigenic site V. **A)** RSB1 and hRSV90 bind site V on opposite sides of helix *α*3, with no apparent overlap in their epitopes. **B)** RSB1 and CR9501 bind highly similar epitopes on RSV PreF. Epitopes for each are mapped onto PreF and colored in cyan, while structures of the Fabs are omitted in this view for clarity.
(PDF)

S9 Fig Germline and sequence comparisons for site V targeting antibodies. **A**) Sequence alignment for LCDR1 and HCDR3 portions of antibodies that contain a Tyr32 in LCDR1. Sequences were obtained from Gilman et al., 2016[30]. For simplicity, only one sequence for each unique heavy and light chain pair was selected. Tyr32_LCDR1_ is shown in bold for LCDR1 sequences. Residues at the tip of the HCDR3 are also in bold. Antibody names are highlighted according to light chain germline groups **B)** X-ray structures of RSB1 and CR9501 Fabs superimposed with homology models for representative antibody light chains from each of the V_L_ gene groups in (A), showing the relative structural alignments of Tyr32. Antibodies are colored according to the highlight scheme in (A).
(PDF)

S1 Table Data collection and refinement statistics. (PDF)

S2 Table Antibody binding affinity for trimeric and monomeric PreF. (PDF)

S1 Data Computational analysis of the RSB1 epitope mutants. (PDF)
