# Supplementary material for: Convergent structural features of respiratory syncytial virus neutralizing antibodies and plasticity of the site V epitope on prefusion F
Source: PLoS Pathog. 2020 Nov 2;16(11):e1008943. doi: 10.1371/journal.ppat.1008943 (PMC7660905; doi:10.1371/journal.ppat.1008943)
Supplement: S1 Fig — (A) Deuterium uptake plots for four peptic fragments of DS-Cav1 alone (blue) and for the complex of DS-Cav1 and RSB1 (red) showing peptides with different deuterium uptake upon complex formation. (B) HDx-MS fragments mapped onto PreF structure. Framgents starting at residues 93 and 199 correspond to portions of site Ø, while fragments starting with residues 57 and 161 correspond to site V. (PDF) [file ppat.1008943.s001.pdf]

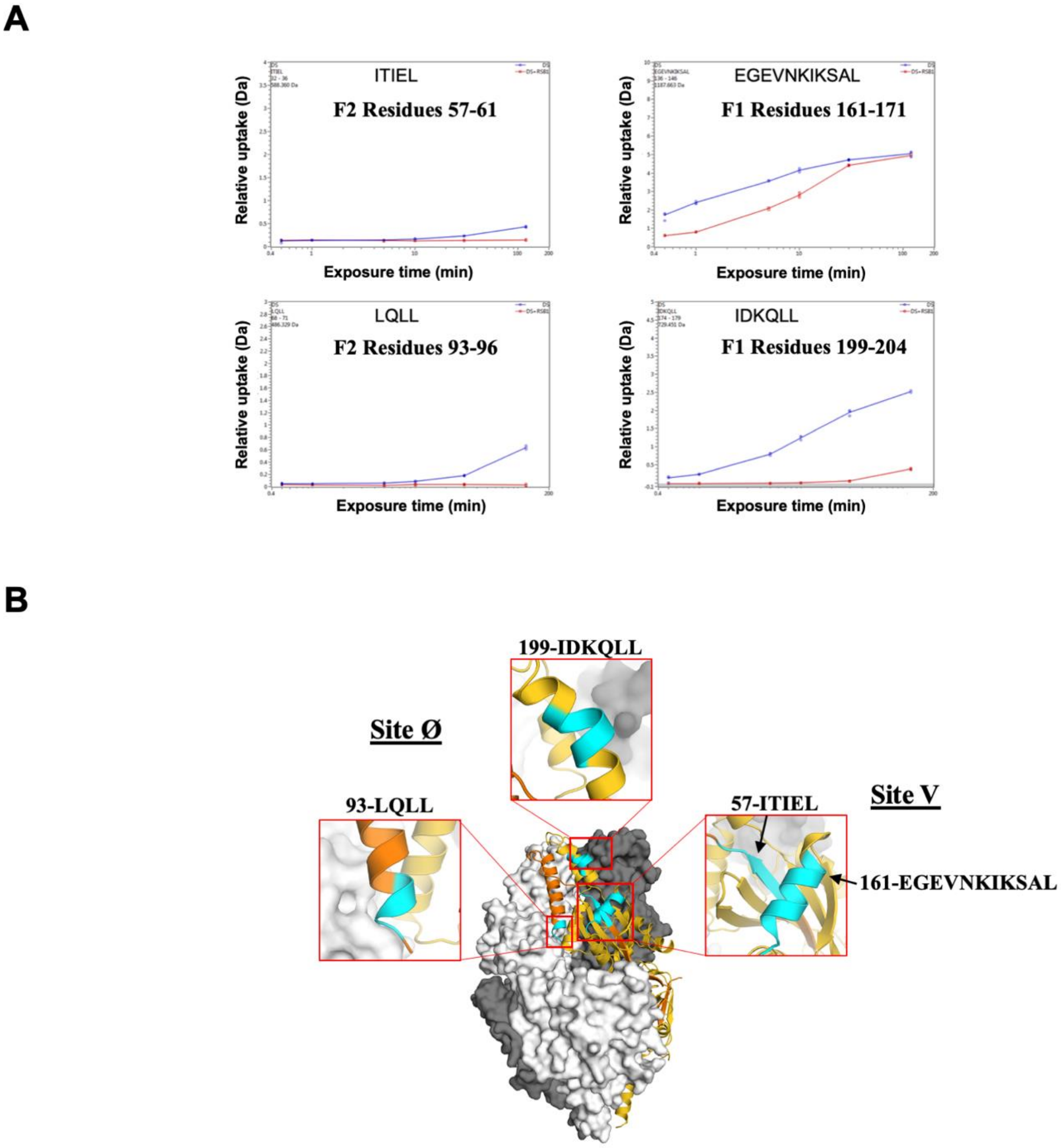

Supplementary Figure 1. Epitope mapping using HDx-MS. **(A)** Deuterium uptake plots for four peptic fragments of DS-Cav1 alone (blue) and for the complex of DS-Cav1 and RSB1 (red) showing peptides with different deuterium uptake upon complex formation. (B) HDx-MS fragments mapped onto PreF structure. Framgents starting at residues 93 and 199 correspond to portions of site Ø, while fragments starting with residues 57 and 161 correspond to site V.
