# Supplementary material for: Convergent structural features of respiratory syncytial virus neutralizing antibodies and plasticity of the site V epitope on prefusion F
Source: PLoS Pathog. 2020 Nov 2;16(11):e1008943. doi: 10.1371/journal.ppat.1008943 (PMC7660905; doi:10.1371/journal.ppat.1008943)
Supplement: S2 Fig — A) 2Fo-Fc electron densities (contoured at 1 sigma) are shown as mesh around one protomer of DS-Cav1 in complex with one RSB1 Fab, as well as zoomed at the heavy chain interface with DS-Cav1. B) 1σ 2Fo-Fc electron densities around the CDRs for the RSB1 Apo structure. (PDF) [file ppat.1008943.s002.pdf]

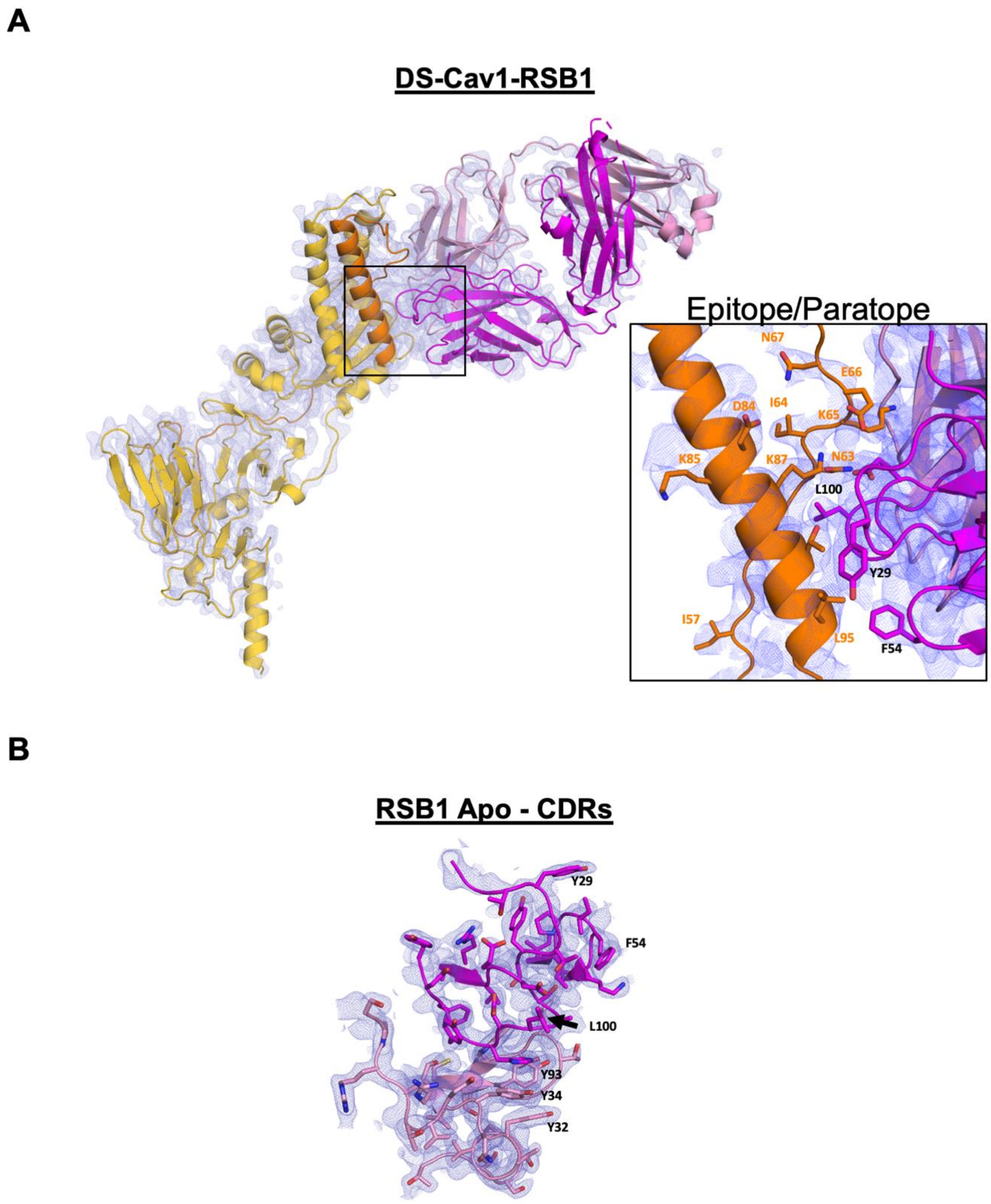

Supplementary Figure 2. A) 2Fo-Fc electron densities (contoured at 1 sigma) are shown as mesh around one protomer of DS-Cav1 in complex with one RSB1 Fab, as well as zoomed at the heavy chain interface with DS-Cav1. **B)** 1σ 2Fo-Fc electron densities around the CDRs for the RSB1 Apo structure.
