# Supplementary material for: Convergent structural features of respiratory syncytial virus neutralizing antibodies and plasticity of the site V epitope on prefusion F
Source: PLoS Pathog. 2020 Nov 2;16(11):e1008943. doi: 10.1371/journal.ppat.1008943 (PMC7660905; doi:10.1371/journal.ppat.1008943)
Supplement: S3 Fig — RSB1 epitope residues defined by X-ray crystallography are clustered on the PreF structure (left, cyan patches). In contrast, in the PostF structure (right), the same residues (cyan) are rearranged in a much more dispersed manner, indicated by black arrows. Distances indicate approximate movement from residue locations in PreF structure. (PDF) [file ppat.1008943.s003.pdf]

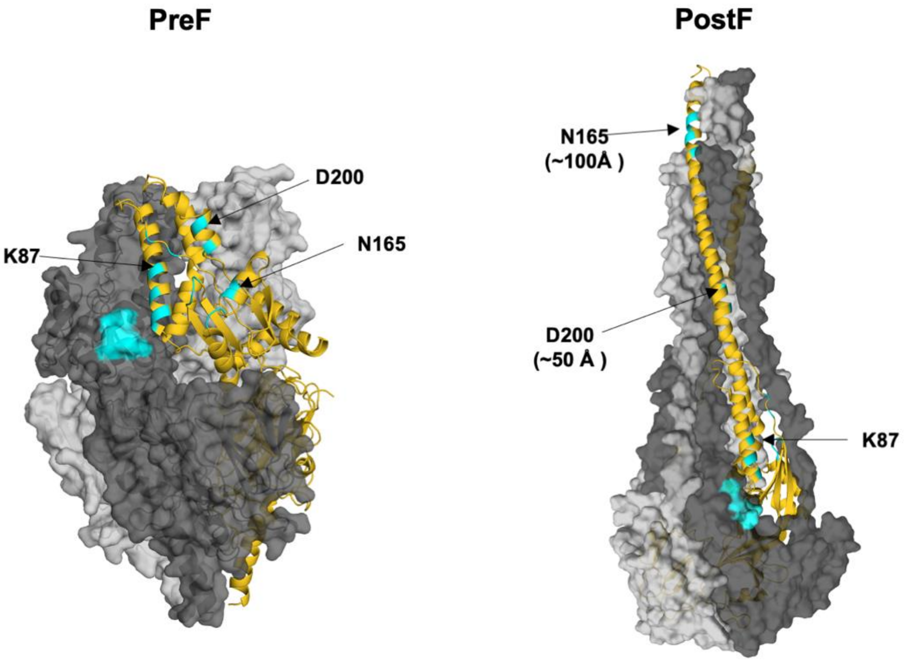

Supplementary Figure 3. RSB1 epitope residues defined by X-ray crystallography are clustered on the PreF structure (left, cyan patches). In contrast, in the PostF structure (right), the same residues (cyan) are rearranged in a much more dispersed manner, indicated by black arrows. Distances indicate approximate movement from residue locations in PreF structure.
