# Supplementary material for: Convergent structural features of respiratory syncytial virus neutralizing antibodies and plasticity of the site V epitope on prefusion F
Source: PLoS Pathog. 2020 Nov 2;16(11):e1008943. doi: 10.1371/journal.ppat.1008943 (PMC7660905; doi:10.1371/journal.ppat.1008943)
Supplement: S4 Fig — RSB1 heavy (magenta) and light (pink) CDRs are shown with cartoon and side chain sticks, while the RSB1 epitope on PreF is shown as surface and colored according to the residue conservation between RSV A and B strains generated using the ConSurf server [61]. Conservation is indicated by a gradient from dark red (highly conserved) to green (not conserved). Residues in the epitope which are not conserved between A and B strains (D200, N276) are labeled in green. (PDF) [file ppat.1008943.s004.pdf]

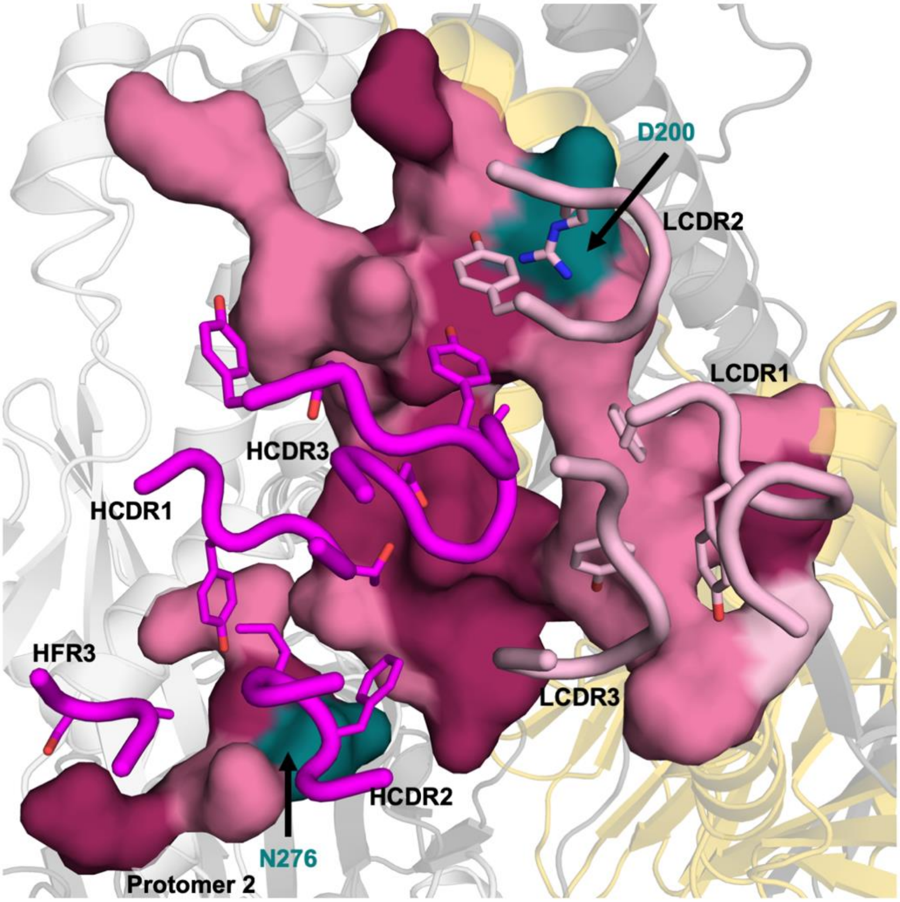

Supplementary Figure 4. RSB1 heavy (magenta) and light (pink) CDRs are shown with cartoon and side chain sticks, while the RSB1 epitope on PreF is shown as surface and colored according to the residue conservation between RSV A and B strains generated using the ConSurf server[1]. Conservation is indicated by a gradient from dark red (highly conserved) to green (not conserved). Residues in the epitope which are not conserved between A and B strains (D200, N276) are labeled in green.
