# Supplementary material for: Convergent structural features of respiratory syncytial virus neutralizing antibodies and plasticity of the site V epitope on prefusion F
Source: PLoS Pathog. 2020 Nov 2;16(11):e1008943. doi: 10.1371/journal.ppat.1008943 (PMC7660905; doi:10.1371/journal.ppat.1008943)
Supplement: S5 Fig — The RSB1 epitope is colored in cyan. Mota heavy chain is colored dark brown and light chain is colored light brown. The Mota Fab bound to the same PreF protomer as a single RSB1 Fab is labeled as Protomer 1, whereas the Mota Fab that competes with RSB1 binding is labeled as the adjacent protomer. The third Mota Fab is not visible from this view. The RSB1 cross-protomer contacts which overlap with the Mota epitope are labeled. (PDF) [file ppat.1008943.s005.pdf]

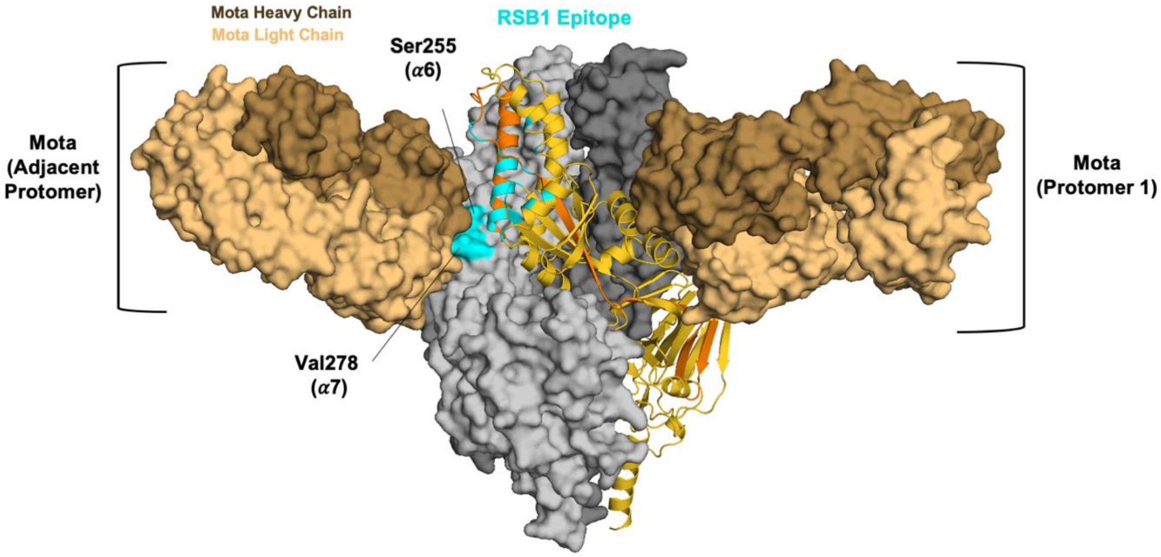

Supplementary Figure 5. Structural basis for competition with Mota. The RSB1 epitope is colored in cyan. Mota heavy chain is colored dark brown and light chain is colored light brown. The Mota Fab bound to the same PreF protomer as a single RSB1 Fab is labeled as Protomer 1, whereas the Mota Fab that competes with RSB1 binding is labeled as the adjacent protomer. The third Mota Fab is not visible from this view. The RSB1 cross-protomer contacts which overlap with the Mota epitope are labeled.
