# Supplementary material for: Convergent structural features of respiratory syncytial virus neutralizing antibodies and plasticity of the site V epitope on prefusion F
Source: PLoS Pathog. 2020 Nov 2;16(11):e1008943. doi: 10.1371/journal.ppat.1008943 (PMC7660905; doi:10.1371/journal.ppat.1008943)
Supplement: S6 Fig — A) Surface potential around PreF residue Asp200 from this study (left), representing RSV A, and the surface potential on RSB1 around the Arg53(LCDR2) which interacts with Asp200 (right). B) Surface potential around PreF residue Asn200 from the structure of RSV B PreF PDB 6Q06 (left), with the RSB1 interacting residue Arg53(LCDR2) is shown again for comparison (right). (PDF) [file ppat.1008943.s006.pdf]

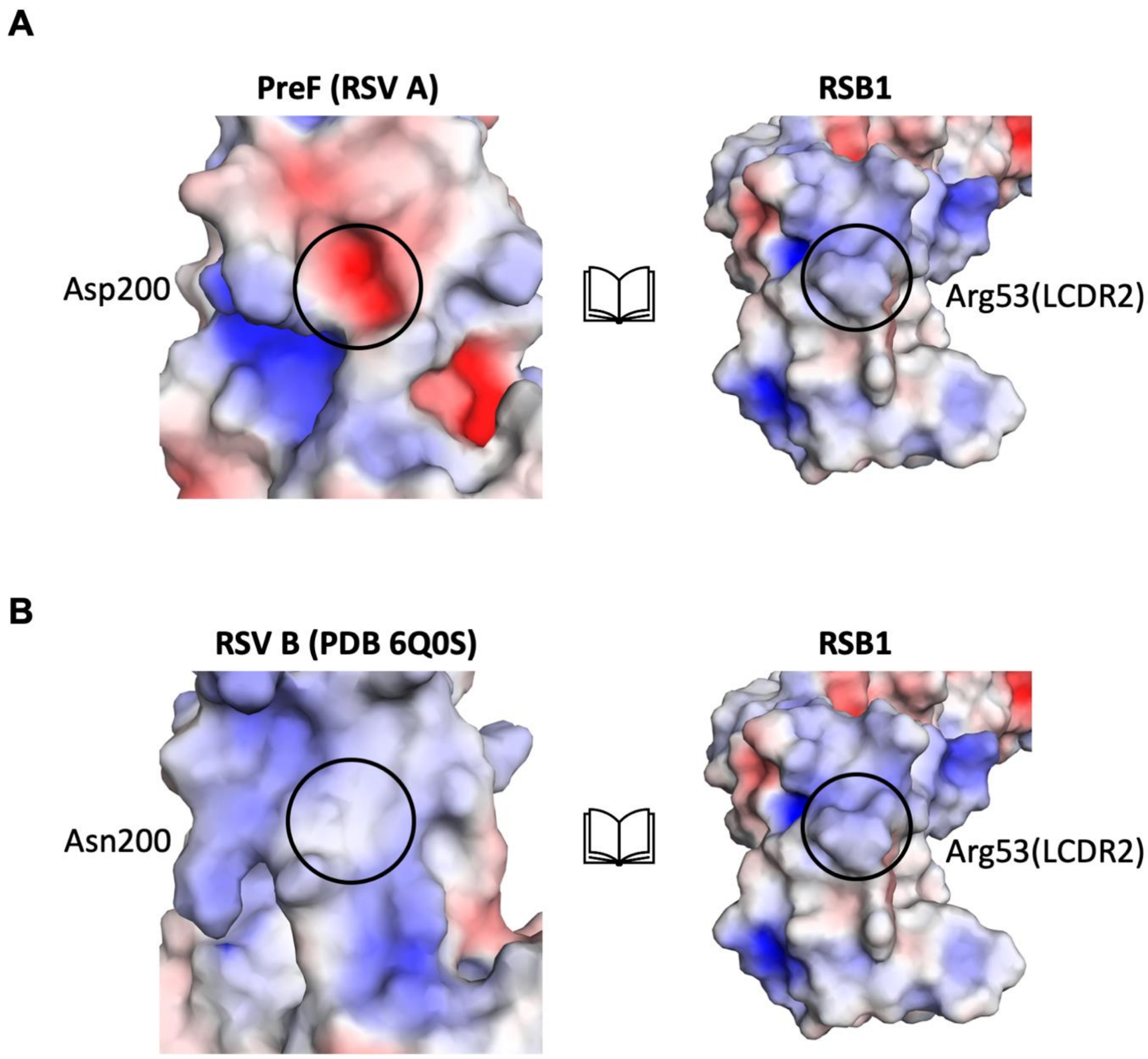

Supplementary Figure 6. Difference in surface potential for PreF residue Asp200/Asn200 in RSV A and B viruses. A) Surface potential around PreF residue Asp200 from this study (left), representing RSV A, and the surface potential on RSB1 around the Arg53$_{(LCDR2)}$ which interacts with Asp200 (right). **B)** Surface potential around PreF residue Asn200 from the structure of RSV B PreF PDB 6Q06 (left), with the RSB1 interacting residue Arg53$_{(LCDR2)}$ is shown again for comparison (right).
