# Supplementary material for: Convergent structural features of respiratory syncytial virus neutralizing antibodies and plasticity of the site V epitope on prefusion F
Source: PLoS Pathog. 2020 Nov 2;16(11):e1008943. doi: 10.1371/journal.ppat.1008943 (PMC7660905; doi:10.1371/journal.ppat.1008943)
Supplement: S7 Fig — A) Comparison of the variable fragments for the RSB1 apo structure (colored white) and the DS-Cav1-RSB1 bound structure (colored as previously). B) Zoomed view of Tyr29HCDR1 which is re-orientated with respect to the unbound state. Interactions with three residues across two DS-Cav1 protomers are shown as sticks with transparent surface. C) Zoomed view of Arg53LCDR2 which is also re-orientated with respect to the unbound state. Salt bridge is shown for interaction with F1 residue Asp200, which is substituted with Asn200 in RSV B viruses. (PDF) [file ppat.1008943.s007.pdf]

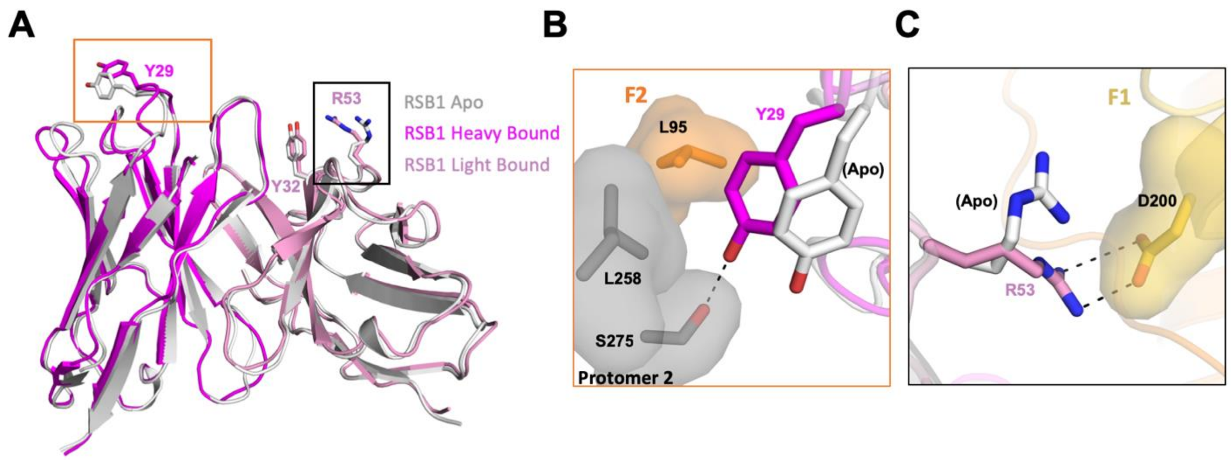

Supplementary Figure 7. RSB1 maintains global conformation between bound and unbound states A) Comparison of the variable fragments for the RSB1 apo structure (colored white) and the DS-Cav1-RSB1 bound structure (colored as previously). **B)** Zoomed view of $Tyr29_{HCDR1}$ which is re-orientated with respect to the unbound state. Interactions with three residues across two DS-Cav1 protomers are shown as sticks with transparent surface. **C)** Zoomed view of $Arg53_{LCDR2}$ which is also re-orientated with respect to the unbound state. Salt bridge is shown for interaction with F1 residue Asp200, which is substituted with Asn200 in RSV B viruses.
