# Supplementary material for: Convergent structural features of respiratory syncytial virus neutralizing antibodies and plasticity of the site V epitope on prefusion F
Source: PLoS Pathog. 2020 Nov 2;16(11):e1008943. doi: 10.1371/journal.ppat.1008943 (PMC7660905; doi:10.1371/journal.ppat.1008943)
Supplement: S8 Fig — A) RSB1 and hRSV90 bind site V on opposite sides of helix α3, with no apparent overlap in their epitopes. B) RSB1 and CR9501 bind highly similar epitopes on RSV PreF. Epitopes for each are mapped onto PreF and colored in cyan, while structures of the Fabs are omitted in this view for clarity. (PDF) [file ppat.1008943.s008.pdf]

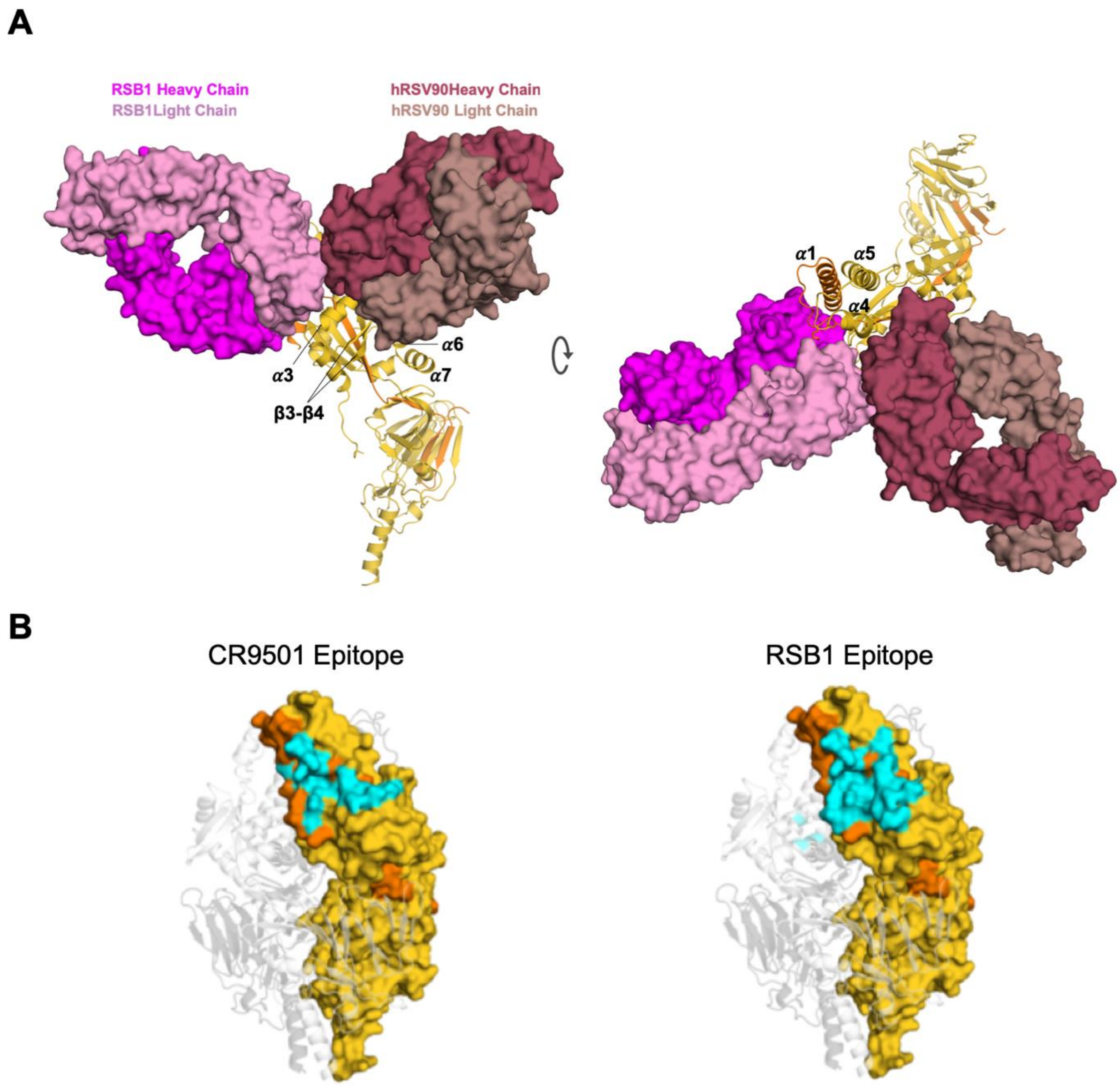

Supplementary Figure 8. Two distinct epitopes at antigenic site V. A) RSB1 and hRSV90 bind site V on opposite sides of helix $\alpha 3$, with no apparent overlap in their epitopes. **B)** RSB1 and CR9501 bind highly similar epitopes on RSV PreF. Epitopes for each are mapped onto PreF and colored in cyan, while structures of the Fabs are omitted in this view for clarity.
