# Supplementary material for: Convergent structural features of respiratory syncytial virus neutralizing antibodies and plasticity of the site V epitope on prefusion F
Source: PLoS Pathog. 2020 Nov 2;16(11):e1008943. doi: 10.1371/journal.ppat.1008943 (PMC7660905; doi:10.1371/journal.ppat.1008943)
Supplement: S9 Fig — A) Sequence alignment for LCDR1 and HCDR3 portions of antibodies that contain a Tyr32 in LCDR1. Sequences were obtained from Gilman et al., 2016[30]. For simplicity, only one sequence for each unique heavy and light chain pair was selected. Tyr32LCDR1 is shown in bold for LCDR1 sequences. Residues at the tip of the HCDR3 are also in bold. Antibody names are highlighted according to light chain germline groups B) X-ray structures of RSB1 and CR9501 Fabs superimposed with homology models for representative antibody light chains from each of the VL gene groups in (A), showing the relative structural alignments of Tyr32. Antibodies are colored according to the highlight scheme in (A). (PDF) [file ppat.1008943.s009.pdf]

A

Antibody	V_L gene	LCDR1	V_H gene	HCDR3
		32		
RSB1	VL2-11	--SGDV-----GT**Y**NY	VH1-69	ARVEDTA**L**--DHY-------FDY
CR9501	VK1-33	---QDI-----ST**Y**--	VH4-31	----AACG--AYVL**I**SNCGWFDS
ADI-18977	VK1-33	QASQGI-----SR**Y**LN	VH1-3	ARQVSTS**G**--WHATSHR---FAP
ADI-14451	VL3-19	HGDTLR-----NY**Y**PA	VH1-2	ARSQQLL**V**ITDYS-------LDY
ADI-15609	VK1-39	RARRSI-----DN**Y**LN	VH3-30	ARDPPAS**A**--AAM-------LDY
ADI-15568	VK1-39	RASQSV-----IT**Y**LN	VH1-46	GREDSYC**S**GDSCFNSGSGRWVDS
ADI-18936	VK1-39	RASQSI-----SG**Y**LS	VH1-2	ASQSSPY**T**--PGA-------MGV
ADI-18966	VK2-30	RSSQSLVYSDGNT**Y**LN	VH3-30	ARDPGVG**S**--YYNVVG----MDV
ADI-14442	VK2-30	RSSQSLVHSDTNT**Y**LN	VH1-18	ARDVPAD**G**--VHF-------MDV
ADI-18975	VK2-24	RSSQSLVHSDGNT**Y**LS	VH1-18	ARDSGCC**S**--GST-------SDV

B

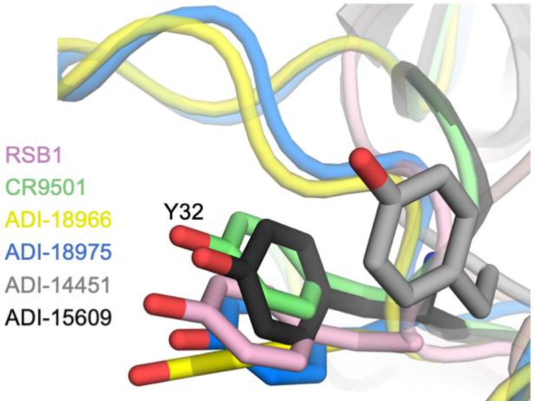

Supplementary Figure 9. Germline and sequence comparisons for site V targeting antibodies. **A**) Sequence alignment for LCDR1 and HCDR3 portions of antibodies that contain a Tyr32 in LCDR1. Sequences were obtained from Gilman et al., 2016[2]. For simplicity, only one sequence for each unique heavy and light chain pair was selected. $Tyr32_{LCDR1}$ is shown in bold for LCDR1 sequences. Residues at the tip of the HCDR3 are also in bold. Antibody names are highlighted according to light chain germline groups **B)** X-ray structures of RSB1 and CR9501 Fabs superimposed with homology models for representative antibody light chains from each of the V_L gene groups in (A), showing the relative structural alignments of Tyr32. Antibodies are colored according to the highlight scheme in (A).
