# Supplementary material for: Convergent structural features of respiratory syncytial virus neutralizing antibodies and plasticity of the site V epitope on prefusion F
Source: PLoS Pathog. 2020 Nov 2;16(11):e1008943. doi: 10.1371/journal.ppat.1008943 (PMC7660905; doi:10.1371/journal.ppat.1008943)
Supplement: S1 Table — (PDF) [file ppat.1008943.s010.pdf]

**Supplemental Table 1. Data collection and refinement statistics.**

| | DS-Cav1-RSB1 | RSB1 |
|---|---|---|
| **Data Collection** | | |
| Wavelength (λ) | 1 | 1 |
| Resolution range | 37.3 - 3.74 (3.8 - 3.7) * | 35.5 - 2.0 (2.07 - 2.0) |
| Space group | $I\ 2_1 3$ | $P\ 2_1$ |
| Cell dimensions | | |
| *a, b, c* (Å) | 190.3, 190.3, 190.3 | 70.4, 82.5, 75.9 |
| α, β, γ (°) | 90, 90, 90 | 90, 95, 90 |
| Total reflections | 134016 (13590) | 194102 (19839) |
| Unique reflections | 11995 (1188) | 55626 (4080) |
| Multiplicity | 11.2 (11.4) | 3.5 (3.6) |
| Completeness (%) | 99.8 (99.8) | 89.1 (70.1) |
| I/σI | 20.79 (3.45) | 14.66 (5.21) |
| Wilson B-factor | 25.69 | 24.41 |
| R-merge | 0.096 (0.690) | 0.205 (0.435) |
| $CC_{1/2}$ | 0.998 (0.919) | 0.843 (0.748) |
| **Refinement** | | |
| Resolution (Å) | 40.6-3.74 | 35.5-2.0 |
| No. reflections | 11986 | 52155 |
| $R_{work}$ / $R_{free}$ (%) | 22/29 | 22/29 |
| No. atoms | | |
| Protein | 6632 | 6401 |
| solvent | - | 581 |
| R.m.s. deviations: | | |
| Bond lengths (Å) | 0.004 | 0.007 |
| Bond angles (°) | 0.76 | 0.95 |
| Ramachandran plot[#] | | |
| Favored (%) | 94.08 | 96.47 |
| Allowed (%) | 5.45 | 3.06 |
| Outliers (%) | 0.46 | 0.47 |
| Average B-factor | 68.03 | 30.15 |
| macromolecules | 68.03 | 30.06 |
| solvent | - | 31.12 |
| Number of TLS groups | 21 | 25 |
| PDB ID | 6W52 | 6W5D |

R.m.s. deviation, root-mean square deviation.
[*]Values in parentheses are for the highest resolution shell.
[#] Measured using Molprobity
