# Supplementary material for: Convergent structural features of respiratory syncytial virus neutralizing antibodies and plasticity of the site V epitope on prefusion F
Source: PLoS Pathog. 2020 Nov 2;16(11):e1008943. doi: 10.1371/journal.ppat.1008943 (PMC7660905; doi:10.1371/journal.ppat.1008943)
Supplement: S2 Table — (PDF) [file ppat.1008943.s011.pdf]

Supplemental Table 2. Antibody binding affinity for trimeric and monomeric PreF

	**RSB1**		**D25**		**AM14**	
	Trimer	Monomer	Trimer	Monomer	Trimer	Monomer
**ka (1/Ms)**	8.06E+05	3.03E+05	1.17E+06	3.84E+05	7.43E+06	--
**koff (1/s)**	1.17E-04	7.93E-05	6.21E-05	3.75E-05	7.70E-04	--
**K_D (M)**	1.47E-10	2.62E-10	5.34E-11	9.77E-11	1.07E-10	--
