# Supplementary material for: Convergent structural features of respiratory syncytial virus neutralizing antibodies and plasticity of the site V epitope on prefusion F
Source: PLoS Pathog. 2020 Nov 2;16(11):e1008943. doi: 10.1371/journal.ppat.1008943 (PMC7660905; doi:10.1371/journal.ppat.1008943)
Supplement: S1 Data — (PDF) [file ppat.1008943.s012.pdf]

Supplementary Methods

Computational analysis of the RSB1 epitope mutants

In order to better understand the dynamics of RSB1 binding to DS-Cav1, we simulated the alanine mutations and RSV B substitution mutations with *in silico* binding affinity prediction tools from the Rosetta Protein Design Suite[3], which calculates interface energies during computational mutagenesis[4,5]. The computed free energies were then compared to binding affinities derived from our experimental SPR data, and a Pearson correlation coefficient was calculated to validate that the experimental and predicted binding energetics were in agreement[5]. A threshold of ± 2 kcal/mol was used to describe stabilizing energetics (ΔΔG or Gibbs free energy < -2 kcal/mol) or destabilizing mutations (ΔΔG > 2 kcal/mol). This threshold was determined based on the standard error between experimental and predicted interface energies (approximately 2.15 kcal/mol as shown in **Figure 7A**). In the case of the alanine mutations Asn63Ala, Lys65Ala in site Ø, this process allowed us to attain a quantitative understanding of the influence on binding of the alanine single mutants, as well as their increased destabilizing role as a double mutant (**Figure 7A**). Specifically, the *in silico* Asn63Ala mutant only slightly destabilized the RSB1 complex, with a ΔΔG of 2.2 kcal/mol. Analyses of pairwise residue interactions suggests that the destabilization is due to two residues in the RSB1 heavy chain; Leu104 which contributes ~0.6 kcal/mol, and Asp105 which contributes ~1.8 kcal/mol. The larger contribution by Asp105 is likely due to the loss of a hydrogen bonding interaction with Asn63 side chain. Additionally, visual inspection of the epitope facing RSB1 Asp105 revealed the presence of a predominant negatively charged patch on PreF, thus suggesting that disruption of electrostatic PreF-RSB1 interactions in this region might result in interface destabilization. The PreF Lys65Ala *in silico* mutation resulted in an even larger predicted destabilization of the RSB1 complex, with a ΔΔG of 3.6 kcal/mol. In this case, pairwise residue interactions show how both the RSB1 light chain (Tyr51: ~0.5 kcal/mol) and heavy chain (Tyr107: ~0.2 kcal/mol, Asp109: ~4.2 kcal/mol) contribute to the destabilization. Interestingly, the Asn63Ala-Lys65Ala double mutant appears to be additive in nature, with an average predicted destabilization energy of ~5.7 kcal/mol. This indicates that the computational analysis may be describing subtle pairwise residue interactions, such as the slight destabilization of the single Asn63Ala mutant, that are beyond the sensitivity limit of the SPR experiments. For D25, no change in binding affinity was predicted in the presence of either single or double mutants, consistent with the experimental SPR data (**Figure 7A**). Finally, the *in silico* mutations on PreF for the RSV B substitutions were predicted to have a negligible change in affinity for both the single mutants Asp200Asn (~1.5 kcal/mol), Asn276Ser (0 kcal/mol), and the double mutant Asp200Asn-Asn276Ser (~1.4 kcal/mol), also in agreement with the experimental SPR data (**Figure 7A**). Collectively, this computational workflow shows that reliable binding affinities can be predicted using Rosetta, while also delivering valuable information on the binding dynamics.
